# The two ends of the spectrum: comparing chronic schizophrenia and premorbid latent schizotypy by actigraphy

**DOI:** 10.1186/s12888-025-06971-5

**Published:** 2025-05-24

**Authors:** Szandra László, Ádám Nagy, József Dombi, Emőke Adrienn Hompoth, Emese Rudics, Zoltán Szabó, András Dér, András Búzás, Zsolt János Viharos, Anh Tuan Hoang, Vilmos Bilicki, István Szendi

**Affiliations:** 1 Doctoral School of Interdisciplinary Medicine, Department of Medical Genetics, University of Szeged, Somogyi Béla Street 4., Szeged, 6720 Csongrád-Csanád Hungary; 2 Department of Software Engineering, University of Szeged, Szeged, 6720 Csongrád-Csanád Hungary; 3 Department of Computer Algorithms and Artificial Intelligence, University of Szeged, Árpád Square 2., Szeged, 6720 Csongrád-Csanád Hungary; 4 HUN-REN-SZTE Research Group on Artificial Intelligence, Institute of Informatics, University of Szeged, Tisza Lajos Boulevard 103., Szeged, 6725 Csongrád-Csanád Hungary; 5 HUN-REN Biological Research Centre, Institute of Biophysics, Temesvári Boulevard 62., Szeged, 6726 Csongrád-Csanád Hungary; 6 Institute for Computer Science and Control (SZTAKI), Center of Excellence in Production Informatics and Control, Hungarian Research Network (HUN-REN), Centre of Excellence of the Hungarian Academy of Sciences (MTA), Kende Street 13-17., H-1111 Budapest, Hungary; 7 Faculty of Economics and Business, John von Neumann University, Izsák Street 10., Kecskemét, 6400 Bács-Kiskun Hungary; 8 Department of Psychiatry, Kiskunhalas Semmelweis Hospital, Dr. Monszpart László Street 1., Kiskunhalas, 6400 Bács-Kiskun Hungary; 9 Department of Clinical- and Health Psychology, Institute of Psychology, University of Szeged, Egyetem Street 2, Szeged, 6720 Csongrád-Csanád Hungary; 10 Centre of Excellence for Interdisciplinary Research, Development and Innovation of the University of Szeged, Dugonics Square 13., Szeged, 6720 Csongrád-Csanád Hungary

**Keywords:** Actigraphy, Mental disease, Machine learning, Disease development

## Abstract

Motor activity alterations are key symptoms of psychiatric disorders like schizophrenia. Actigraphy, a non-invasive monitoring method, shows promise in early identification. This study characterizes Positive Schizotypy Factor (PSF) and Chronic Schizophrenia (CS) groups using actigraphic data from two databases. At Hauke Land University Hospital, data from patients with chronic schizophrenia were collected; separately, at the University of Szeged, healthy university students were recruited and screened for PSF tendencies toward schizotypy. Several types of features are extracted from both datasets. Machine learning algorithms using different feature sets achieved nearly 90-95% for the CS group and 70-85% accuracy for the PSF. By applying model-explaining tools to the well-performing models, we could conclude the movement patterns and characteristics of the groups. Our study indicates that in the PSF liability phase of schizophrenia, actigraphic features related to sleep are most significant, but as the disease progresses, both sleep and daytime activity patterns are crucial. These variations might be influenced by medication effects in the CF group, reflecting the broader challenges in schizophrenia research, where the drug-free study of patients remains difficult. Further studies should explore these features in the prodromal and clinical High-Risk groups to refine our understanding of the development of the disorder.

**Supplementary Information:**

The online version contains supplementary material available at 10.1186/s12888-025-06971-5.

## Introduction

Alterations in motor activity are critical in diagnosing major psychiatric disorders, including schizophrenia, depression, bipolar disorder, ADHD, anxiety disorders, and autism spectrum disorder (ASD). These alterations often manifest as disruptions in sleep and circadian rhythms, significantly affecting patient outcomes [1, 2].

In conditions such as schizophrenia, bipolar disorder, depression, and ASD, patients commonly exhibit a decrease in overall activity levels. This decrease is associated with poorer sleep quality, fragmented sleep, and reduced sleep duration, all of which correlate with symptom severity [3, 4]. Furthermore, disruptions in sleep patterns, such as reduced sleep duration and negative dream content, are known predictors of symptoms like paranoia [5].

Recent studies using actigraphy have revealed distinctive patterns in schizophrenia patients compared to healthy controls, including lower average activity levels and irregular sleep patterns. These patterns significantly influence key aspects of daily living, such as sleep, social functioning, and emotional stability, demonstrating the profound impact of disturbed rest-activity rhythms on the disorder [6, 7].

The concept of premorbid latent schizophrenia refers to a broad and not well-defined vulnerability that precedes the onset of schizophrenia and lacks established diagnostic criteria. To operationalize this concept, researchers often focus on measurable aspects like positive schizotypy, which includes cognitive and perceptual traits similar to schizophrenia, such as magical thinking, unusual perceptions, and paranoid attitudes [8, 9]. However, identifying premorbid markers is challenging due to their variability and low specificity, which can lead to unnecessary stigma and treatment exposure for individuals who may not develop schizophrenia [9, 10]. Despite these challenges, early identification and intervention are crucial for improving outcomes, as they can delay or mitigate the onset of psychosis [9].

Actigraphy, a non-invasive method that assesses limb movement, has emerged as an essential tool for the objective evaluation of physical activity and sleep patterns. This technique is crucial not only for diagnostic purposes but also for monitoring the efficacy of therapeutic interventions in real-time. Actigraphy metrics such as intradaily variability and interdaily stability provide valuable insights into an individual’s circadian rhythm, offering a reliable measure of treatment impacts and symptom fluctuations [11–14].

The previous research study conducted by our research group found that actigraphy measures and machine learning can be used to identify pre-existing traits associated with schizotypy and bipolarity. A wrist-worn device collected data from healthy participants in three groups: Control Group (C), Cyclothymia Factor Group (CFG), and Positive Schizotypy Factor Group (PSF). We extracted relevant metrics and employed machine learning algorithms for categorization. The results provided insights into important traits for detecting bipolarity and schizotypy. This approach may enable cost-effective and automated early detection of individuals at risk for mental disorders [15].

In this study, we utilize innovative data analysis methods to solve significant challenges. Our analysis employs two advanced machine-learning frameworks: Adaptive Hybrid Feature Selection (AHFS) [16] and Clique Forming Feature Selection (CFFS) [15, 17]. AHFS dynamically combines correlation-based and information-theoretic methods to achieve higher accuracy in high-dimensional datasets. CFFS finds potentially useful feature sets and then calculates feature importance using Shapley values aggregated across multiple ML models with reduced inter-correlation between features. AHFS identifies compact, high-performing feature subsets while CFFS identifies a broader set of globally important features, providing complementary approaches for robust feature selection with maintained interpretability.

Our study has two goals. The first is to distinctively characterize Positive Schizotypy Factor Group (PSF), and Chronic Schizophrenia (CS) subject groups using specific actigraphic features. Each Schizotypic group was compared to its Controls. The second is to increase the sensitivity and specificity of actigraphic data interpretation for detecting early signs of schizotypal development, thus enabling the timely identification and treatment of individuals at risk. The present study is a continuation of our previously published research on the subject [15], further developing the methods used to better understand the actigraphy patterns of the two datasets, which may allow a deeper understanding of the mechanism of the disorder.

## Methods

### Participant selection - Haukeland University Hospital Dataset

The CS group in the present study consists of the psychiatric patient population from the Haukeland University Hospital Dataset (HUHD), sourced from a study by [13], which included 23 chronic schizophrenia patients, 23 individuals currently experiencing depression, and 32 healthy controls. The CS group, predominantly male (19 males, 3 females), had an average age of 46.2 years (range 27–69 years). All participants were receiving antipsychotic medications and were diagnosed during stable phases of chronic illness. The depression subset included five inpatients. The mean age at first hospitalization across the patient groups was 24.8 years (range 10–52 years). The Control Group comprised 20 females and 12 males with a mean age of 38.2 years (range 21–66 years). All participants were assessed using DSM-IV criteria through semi-structured clinical interviews. In the present study, only data from the CS group and the Control group were used, and data from individuals living with depression were not included in any of the analyses.

In the CS group, patients received their first hospital treatment, on average, at the age of 24.4, including cases of childhood onset. Thus, they have been living with chronic schizophrenia for approximately 20 years. All patients lived in open, long-term care facilities as they were unsuitable for independent living despite their stable illness phase. The majority (87%) were male, a demographic associated with a less favorable disease course. All were under antipsychotic treatment, with nearly 40% receiving clozapine, primarily indicated for therapy-resistant cases with poor prognoses requiring institutional care. Patients on clozapine exhibited more pronounced persistent symptoms, even during stable phases. Others received either atypical or conventional antipsychotic treatment.

The original study found reduced total and nocturnal activity, with increased rest-activity cycle regularity, especially in those taking clozapine. However, it did not systematically examine medication side effects (including extrapyramidal effects commonly associated with antipsychotics) or provide details on individual drug types and dosages. Long-term antipsychotic use can lead to Parkinsonism, associated with decreased movement, reduced complexity, and stereotyping-patterns observed in this patient group. The study also did not address correlations between negative symptoms and actigraphy indicators. Negative symptoms can limit psychomotor behavior alongside cognitive dysfunctions, reducing spontaneous movements, expressive gestures, and affective reactions. Additionally, the institutional environment likely influenced motor behavior through monotonous, stereotypical routines. Our analysis identified comprehensive decreases in movement quantity and variability in these chronic patients, likely related to their severe underlying disease, symptom patterns, living conditions, and potential medication side effects.

### Participant selection - University of Szeged Dataset

The University of Szeged Dataset (USD) was made by screening university students, involved self-report questionnaires: the TEMPS-A for assessing affective temperaments and the shortened O-LIFE for subclinical schizotypal traits [18, 19]. Out of 710 screened students, 182 met the inclusion criteria. After initial screening, participants completed the Clinician Version (SCID-CV) of the Structured Clinical Interview for DSM-5 to identify current or former psychiatric disorders. Exclusion criteria included current psychiatric disorders, high mood disorder risk (positive MDQ criteria), substance abuse, history of head trauma with permanent loss of consciousness, and physical illnesses known to affect brain structure or function. Further details in Table 1.

**Table 1:** Participant selection criteria and final sample description for the University of Szeged dataset

Selection criteria	Details
Initial Inclusion	First- and second-year university students aged 18–25 years without previously diagnosed psychiatric disorders.
Screening Tools	TEMPS-A (Affective temperament), O-LIFE (Schizotypy), PDI-21 (Delusional ideation), MDQ (Mood disorder risk).
Eligible after Screening	182 students met the initial inclusion criteria based on questionnaire scores.
Exclusion Criteria	Presence of current psychiatric disorders (SCID-5), high mood disorder risk (MDQ criteria positive), substance abuse, neurological or severe physical illness affecting brain function.
Final Group Criteria	PSF Group: O-LIFE > 5, PDI-21 > 10, TEMPS-A Cyclothymia < 12. (*N* = 26)
	Control Group: O-LIFE < 6, PDI-21 < 11, TEMPS-A Cyclothymia < 12. (*N* = 29)
Data Quality Control	Actigraphy data manually inspected; removal of days with incorrect device usage or device malfunction.
Final Sample Size Analyzed	PSF Group: N = 22 (11 men, 11 women), mean age 26.20 (SD = 2.06).
	Control Group: N = 25 (11 men, 14 women), mean age 25.42 (SD = 1.90).

Participants were screened for schizotypal and cyclothymic traits. The Positive Schizotypy Factor (PSF) group exhibited elevated schizotypal traits (high O-LIFE and PDI-21 scores) and normal cyclothymia scores. The control group showed low scores across all psychopathological measures, indicating no significant psychopathological traits. Cyclothymia group data were not used in this study

After applying all exclusion criteria, 55 students remained, split into:

- **PSF Group**: Participants aged 18–25 with a PSF tendency toward schizotypy, marked by specific scores on the PDI-21 (total score> 10) and O-LIFE (total score > 5), but lower scores on TEMPS-A Cyclothymia (< 12).
- **Control Group**: Participants of the same age range with average scores on every questionnaire [15] (TEMPS-A Cyclothymia < 12, PDI-21 < 11, and O-LIFE < 6).

The final cohorts included 26 in the PSF group and 29 controls. Actigraphic data were successfully collected from 47 individuals (22 men and 25 women). The PSF group consisted of 22 participants, and the control group included 25 participants. Most participants (80%) took no medications; 14% took birth control pills, 3% took beta blockers, and 3% took antihistamines. Device malfunctions or incorrect usage reduced the initial number. All subjects gave written informed consent following the Declaration of Helsinki. The study was approved by the Human Investigation Review Board of the University of Szeged (No. 267/2018-SZTE). Device malfunctions or incorrect usage reduced the initial number. Participant demographics and further selection details are available in Supplementary 1.2. In the present study, we analyzed the CS and PSF groups along with their respective control groups, excluding data from individuals with depression. Each group (CS and PSF) was compared separately to its control group. However, a direct comparison between the CS and PSF groups was conducted only based on SHAP scores.

### Devices

For the Haukeland University Hospital Dataset, the Actiwatch device (model AW4) utilized a piezoelectric accelerometer that captured movements with a sampling frequency of 32 Hz and a resolution of 0.05 g. The device integrated movement intensity, amount, and duration across the x, y, and z axes. Data were filtered in the 3–11 Hz frequency range and stored as activity counts per minute. Sleep periods longer than 15 hours were considered invalid and removed from analysis, as they likely represented device malfunctions.

The University of Szeged dataset used a compact, custom-developed sensor by [20], (41 mm $\times$ 16 mm $\times$ 11.3 mm, 5.94 g) with a microcontroller (C8051 F410), a 3-axis accelerometer (LIS3DH), 1 GB flash memory, and a quartz clock (accuracy: ±20 ppm). This customizable sensor records at 10 samples per second, allowing for weeks of continuous measurement. After manual inspection, days with device failures or incorrect usage were removed from the analysis.

Table 2 gives a comparison of the two devices. The developed actigraphy sensors were used to record triaxial data. Due to the lack of access to raw data in the Norwegian dataset, preprocessing adhered to the Actiwatch AW4 manual to match the PIM method used in the original study. Maximum values per second were summed over one-minute epochs. Full details of the preprocessing steps and the associated codes are documented in the Supplementary Materials *codes* folder and are detailed in Supplementary 4.

**Table 2:** Comparison of actigraphy sensors used in the Norwegian and Szeged datasets

Specification	Actiwatch AW4 (Haukeland University Hospital Dataset)	Custom-Built Sensor (University of Szeged Dataset)
Manufacturer	Cambridge Neurotechnology Ltd, England	University of Szeged (Custom-developed)
Sensor type	Piezoelectric accelerometer	LIS3DH 3-axis accelerometer
Sampling frequency	32 Hz	10 Hz
Recording duration	Continuous, stored in memory	Continuous, stored in flash memory (up to weeks)
Data filtering	Band-pass filter (3–11 Hz)	Butterworth band-pass (0.25–2.5 Hz)
Aggregation method	Activity counts per minute epoch	Maximum per second, integrated per minute epoch (PIM method)
Data validity criterion	Sleep periods $\le$ 15 hours/day	Manually reviewed, corrupted data removed

The Actiwatch AW4 is a commercial device, whereas the custom-built sensor was specifically designed for research at the University of Szeged

**Fig. 1:**
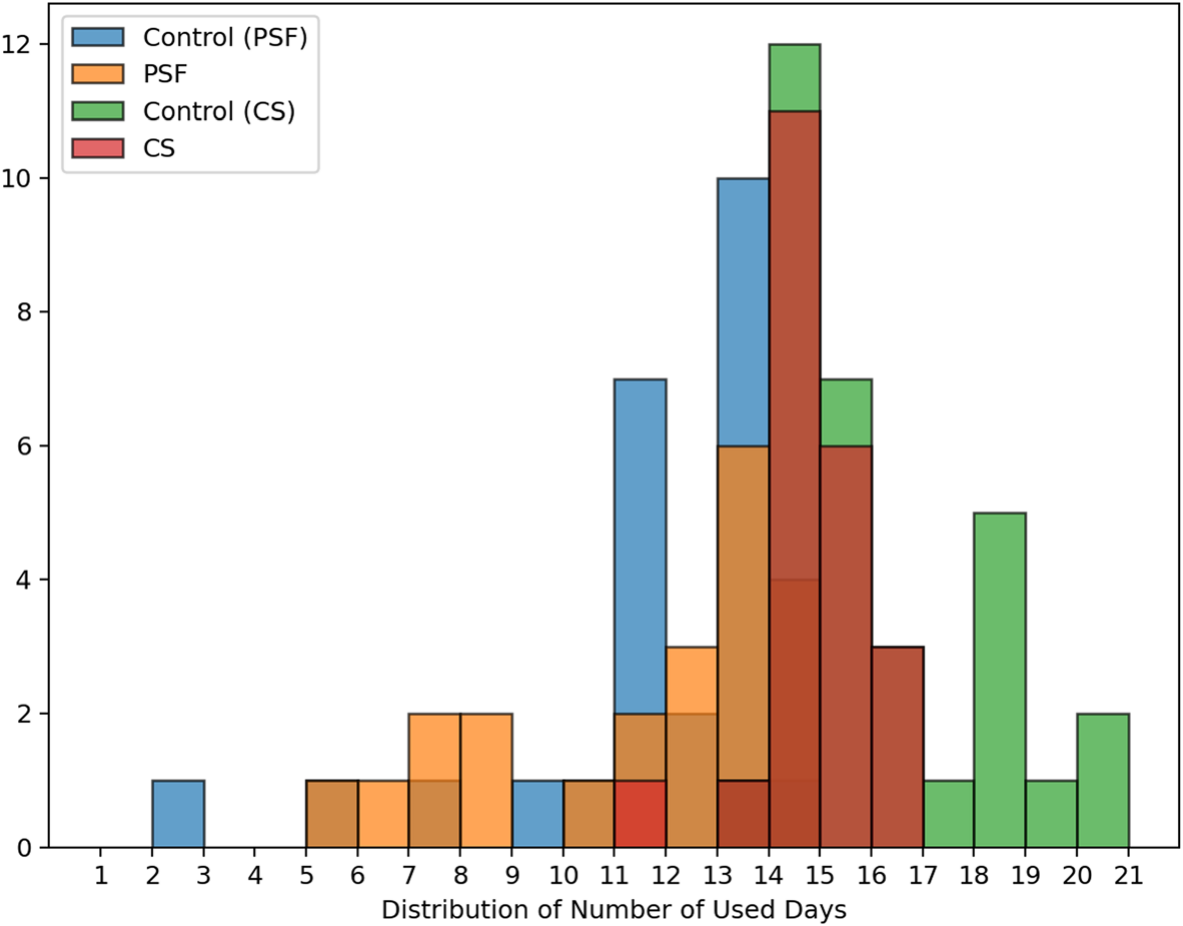
Remaining numbers of the days of each participant after data filtering. The Norwegian dataset is represented with colors of red (CS) and green (Control), and the Szeged dataset with orange (PSF) and blue (Control)

After all of the preprocessing, the remaining numbers of the used day per person are shown in Fig. 1. Days with entirely missing signals (e.g., device shut-off or falling from the wrist) were excluded from the analysis. Those sections are typically presented as zero-value data over a long period (days). No partial-day exclusion criteria were applied, as missing data typically affected full days rather than short periods.

### Feature engineering

We computed usual characteristics researched in mental disorders using the pyActigraphy library [21], a popular, open-source Python package developed specifically for actigraphic data processing. The resulting features, as shown in our previous work [15], comprised measures commonly employed in the literature to evaluate susceptibility to mental illness. The most active 10-hour mean (M10), the least active 5-hour mean (L5), and interdaily stability (IS) were notable characteristics. Table 3 lists the six features of this nature that were developed in total.

**Table 3:** Features from the pyActigraphy package

Feature	Description
M10	Daily mean activity of the 10 most active hours
L5	Daily mean activity of the 5 least active hours
RA	Relative rest/activity amplitude.
ADAT	Total average daily activity
IS	Interdaily stability
IV	Intradaily variability

Other features were calculated from the data, like “activity_mean”, “activity_std” (mean and standard deviation of the activity data), “zero_ratio”, and FI_m and FI_std (mean and standard deviation fragmentation index of every sleep). The fragmentation index is a widely used global measure for assessing sleep quality. It quantifies the proportion of time spent on mobile activities during sleep, including periods of high activity and short passive intervals, relative to the total sleep duration from falling asleep to waking up. Elevated fragmentation index values are often associated with a variety of mental or physical disorders. However, this measure does not provide information about the distribution of nocturnal awakening periods.

#### Sleep movement characteristic

**Fig. 2:**
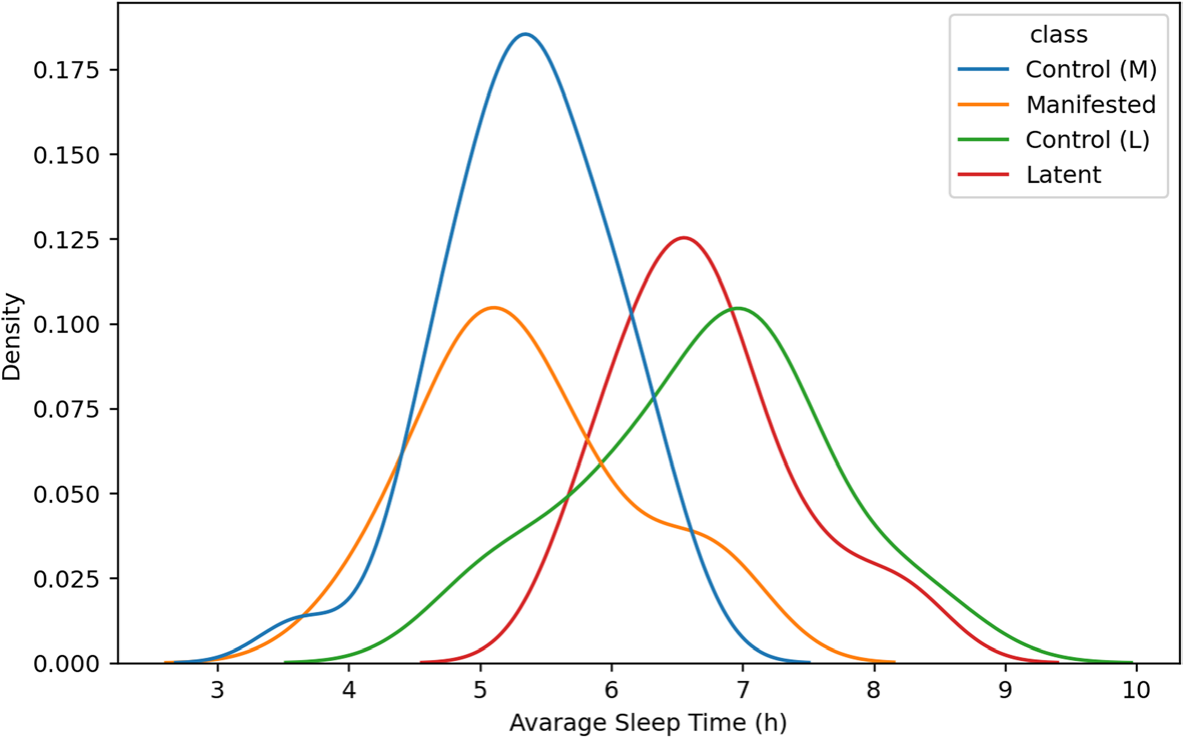
Distribution of the detected sleep time, broken down by groups

Building upon our previous research [15], we have generated features that capture activity patterns during sleep. Since daytime activity highly varies from person to person, even day to day, we analyzed nocturnal movement. Different characteristics were generated to obtain detailed information about participants’ sleep movements. To enhance the sleep detection algorithm, we devised a solution that incorporates a threshold defining prolonged periods of low activity and also adjusts segments shorter than a specified duration (representing sleep or wakefulness) to the opposite state. This approach allows for a more comprehensive characterization of sleep states based on activity levels. The fine-tuning of the parameters was decided based on a visual inspection of the data and the distribution of the average sleep time of the studied groups, as can be seen in Fig. 2.

**Fig. 3:**
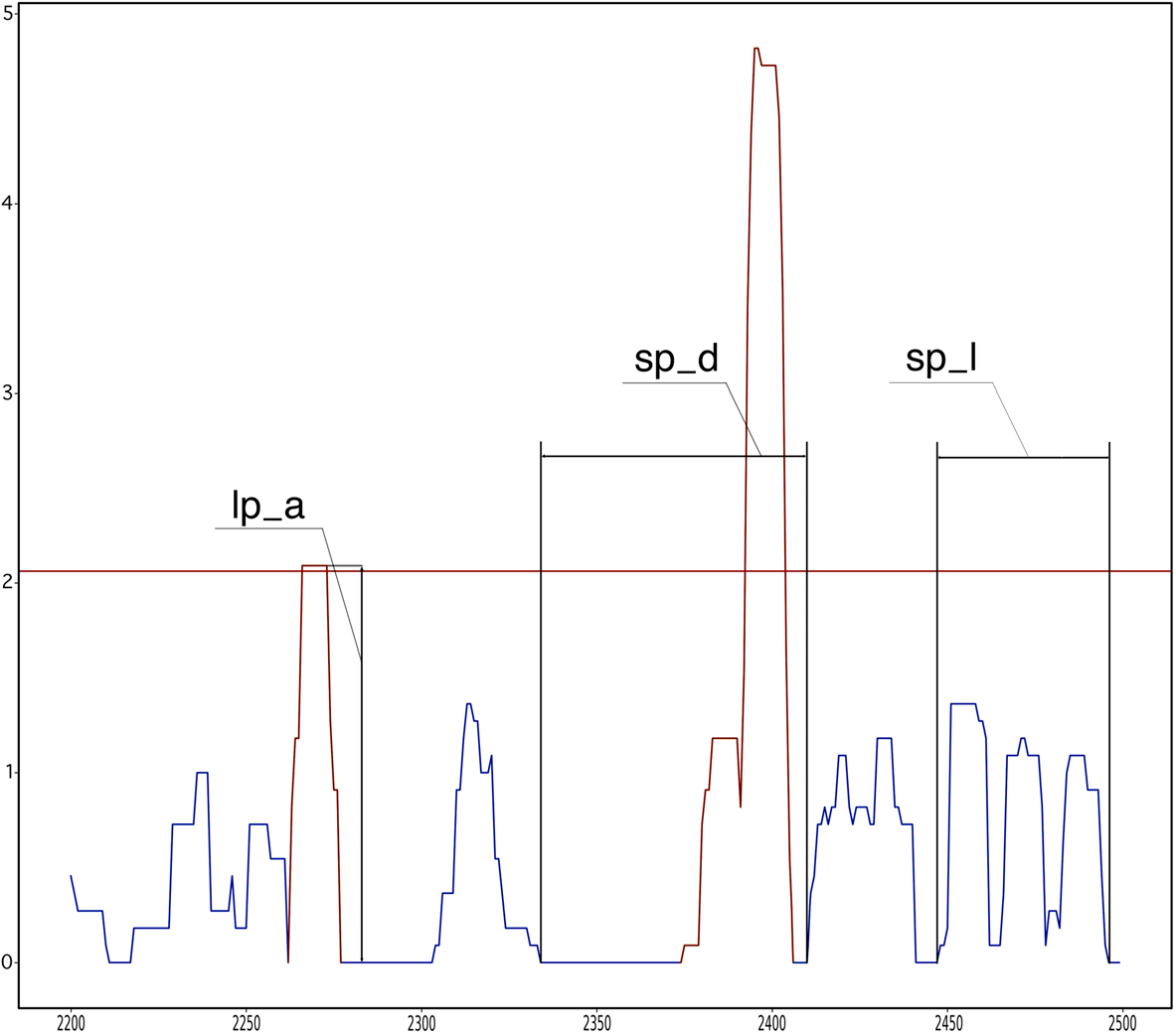
Slice of the activity data with sleep movement features. Horizontal line is the median/upper quartile boundary

The features extracted from this data during our earlier research [15] characterized various “peak” characteristics. The extracted features are displayed in Table 4 and Fig. 3.

**Table 4:** Extracted features categorized by type

Peak categories	Peak metrics	Functions
p_ (“peaks”)	_a (amplitude)	avg() (average)
lp_ (“large peaks”)	_l (length)	std() (standard deviation)
sp_ (“small peaks”)	_d (distance)	max()
(lp/sp)_q (quartile separation)	_cls (close peaks)	min()
_first_ (first peak at the given night)		med() (median)
_last_ (last peak at the given night)		nbr() (number of the given peaks)

A “peak” (p_) in the curve indicates activity data greater than 0, and zero values separate two peaks. The peaks were categorized as small (sp_) or large (lp_), and various characteristics were calculated, including the distance (_d), length (_l), and amplitude (_a) between them. This allowed us to derive characteristics such as minimums, maximums, means, and medians. Small and large peaks were distinguished in two ways: along the median and by the lower and upper quartiles. Each characteristic sleep movement feature was calculated for each night, with separators for large and minor peaks, and then averaged for each individual

#### Wavelet

**Fig. 4:**
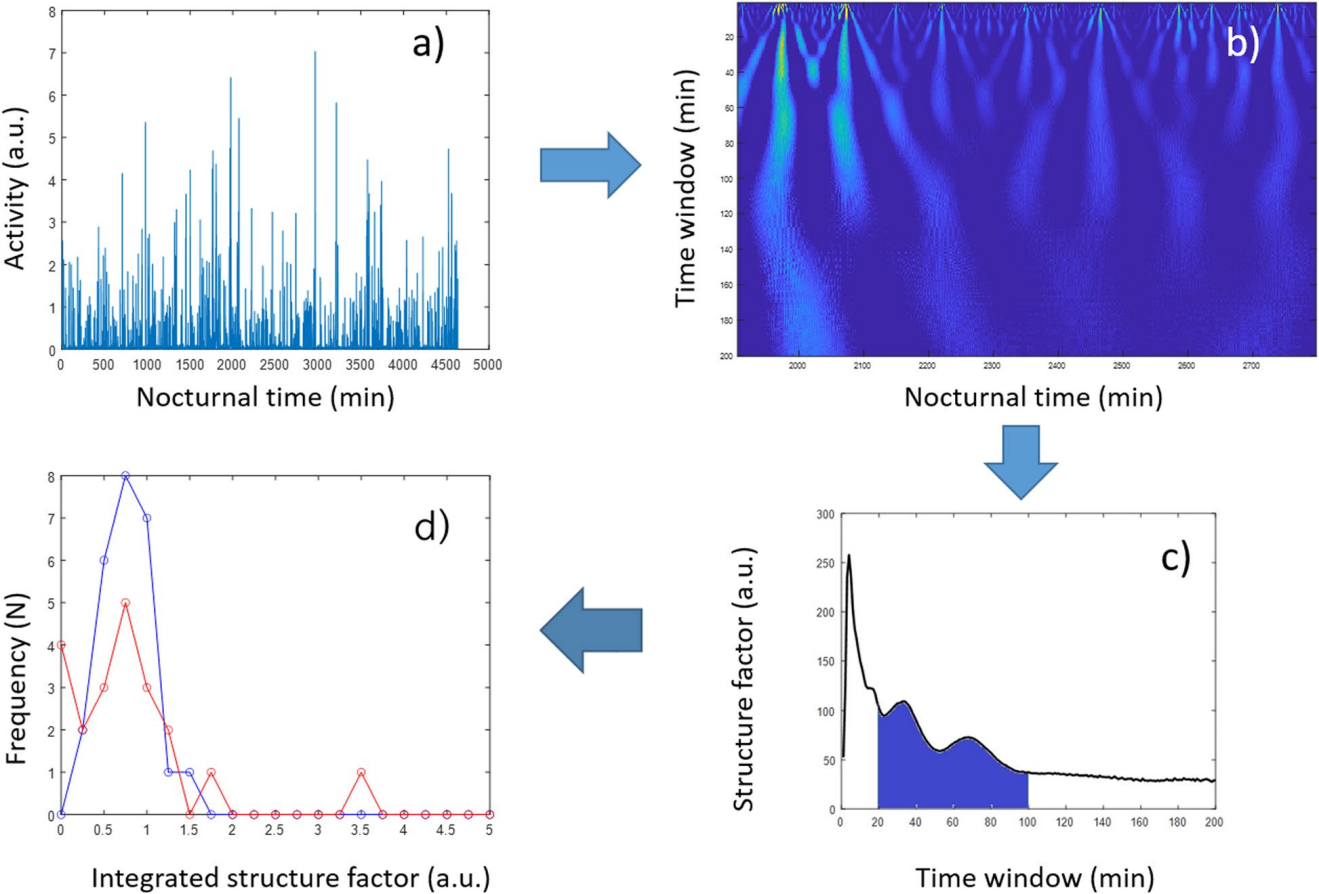
Illustration showing the wavelet-based analysis of nocturnal activity structures. **a** Activities of concatenated sleep periods for five consecutive nights. **b** Correlation-coefficient map of (**a**)’s time series, as determined by continuous wavelet analysis. **c** Structure parameters derived from the map in (**b**), as a function of the scale parameter for the 1–200 s time window. **d** Distribution of integrated structure factors (structure_pms) across the two volunteer groups (Control Group (blue) and Positive Schizotypy Factor Group (red))

In our analysis, as described in our previous work [15, 22] we employed the Morlet wavelet as the MW, which is a single-frequency sinusoidal wave enveloped by a Gaussian function. The Morlet wavelet on actigraphy data (Fig. 4a), also known as the Gabor wavelet, provides a favorable balance between time and frequency resolution [23] and has been utilized effectively in the analysis of physiological signals [24, 25].

By applying Continuous wavelet transform (CWT) with the Morlet wavelet, we aimed to extract crucial information about the dynamic behavior of the biological system and to identify regions of the time series with prominent structural features, as indicated by high amplitude correlation coefficients in the 2D map (as depicted in Fig. 4b). The exact execution and further details about feature processing are presented in the Supplementary 1.3.1.

### Feature selection

#### Shapley values

Originally used as a proven proposition in game theory, Shapley values determine the contribution of each participant to the outcome of a game. In the context of machine learning, Shapley Additive Explanations (SHAP) [26] applies Shapley values to effectively identify the features with the greatest impact on decision-making.

In our analysis, we use a Summary plot to visualize the impact of each feature on the model’s decisions. Each dot on the plot represents the effect of a feature for one individual in the dataset, plotted along the x-axis. A dot positioned further to the right suggests that the feature pushes the model towards classifying the individual as more likely to develop a disease. Conversely, a dot on the left implies a push towards a less likely classification. The position and color of each dot reflect the feature’s value and its influence on the prediction, respectively. For example, lower values in features like mean and standard deviation of daily activity are color-coded to show a higher risk of bipolarity. This visualization helps us understand how different features affect the model’s predictions across all individuals in the study. The SHAP and its use in-depth description available in Supplementary 1.4.1.

#### CFFS (Clique Based Feature Selection)

The feature selection procedure, previously presented in [15], was further refined and improved in our study. CFFS uses machine learning algorithms as well to select features. Due to the small datasets and lack of pretrained larger models, we are limited to simple machine learning algorithms [27]. In this research, we used the Logistic regression (LR) [28], Random Forest (RF) [29], and Artificial Neural Network (ANN). In the following, we want to compare CFFS and AHFS. Since AHFS works in a MATLAB environment and is less malleable, we have tried to integrate the neural network used there into CFFS using Python. However, due to the differences between the two programming languages, this could only be partially done. ANN will serve as a basis for comparing the two methods. The CFFS method is further detailed in Supplementary 1.4.2.

**Fig. 5:**
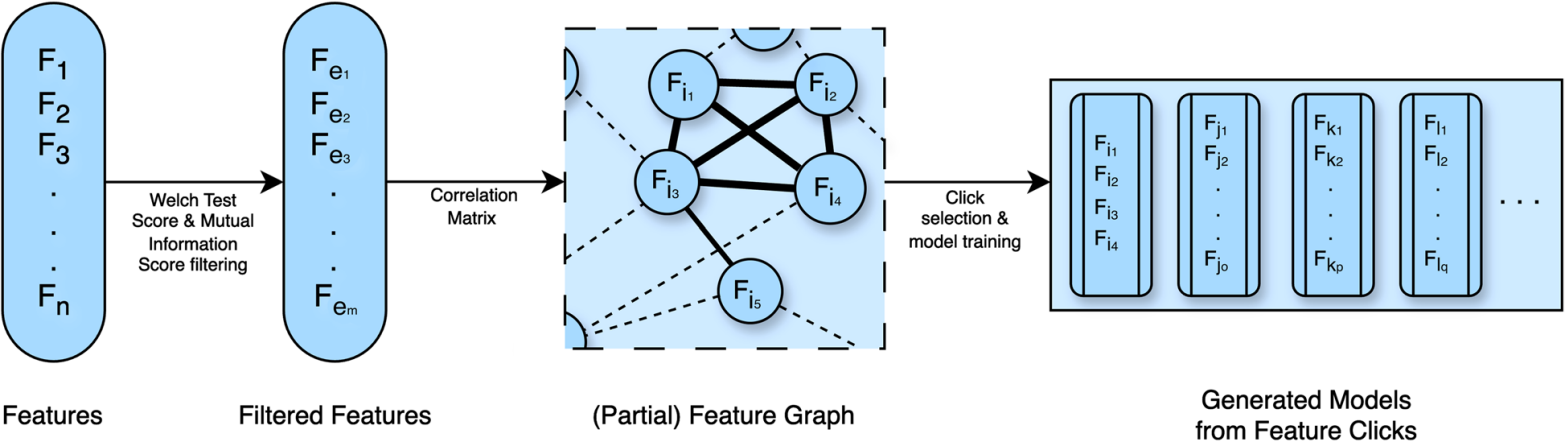
The CFFS steps are presented in a flowchart, which *F* represents the features. It begins by selecting features based on Welch’s test for statistical significance and mutual information, choosing only those that meet specific criteria for both. It then constructs a complete weighted graph using pairwise Pearson correlations among the selected features ($F_{e_1-e_m}$ is a feature subset of $F_{1-n}$ full feature set). Edges exceeding a certain correlation threshold are deleted to reduce inter-feature correlation. The process concludes by identifying cliques within this graph, which represent optimal combinations of less intercorrelated features. For example, $F_{i_1-i_4}$ is a 4-element whole subgraph (Clique) and will be a feature candidate; however, $F_{e_5}$ was left out from the analysis because it does not have enough low correlation features. Those Clique were used for training machine learning algorithms with a focus on maximizing accuracy and interpretability through Shapley values in a 3-fold cross-validation framework

The feature set selection and model-making schematic are shown on Fig. 5. Then the models were filtered with a minimum accuracy of 60%. By aggregating the corresponding Shapely values columns from each selected model, a comprehensive understanding of the model’s performance within the group was obtained. This novel aggregation process ensures the elimination of individual outliers and provides a robust depiction of the models’ functionality. This analysis give a board overview. The utilized machine learning algorithms and all of their parameters, and the exact feature selection algorithm are provided in the Supplementary Materials, the *ML_SHAP.py* python script, which is located in the *codes/ML folder*.

#### AHFS

The Adaptive, Hybrid Feature Selection (AHFS) algorithm [16] introduces a hybrid and adaptive approach to feature selection [30] for machine learning. It combines existing supervised feature selection techniques, each with its own specific evaluation measures, to create a versatile solution. The algorithm incorporates the most frequently used correlation and information-theoretic-based measures, such as MMIFS [31], mRMR [32], LCFS [33], and JMI [34, 35], etc., to assess the relationship between features and their information content. This hybridity enables the integration of additional feature selection methods and metrics, making the proposed algorithm adaptable to different scenarios and future scientific results.

**Fig. 6:**
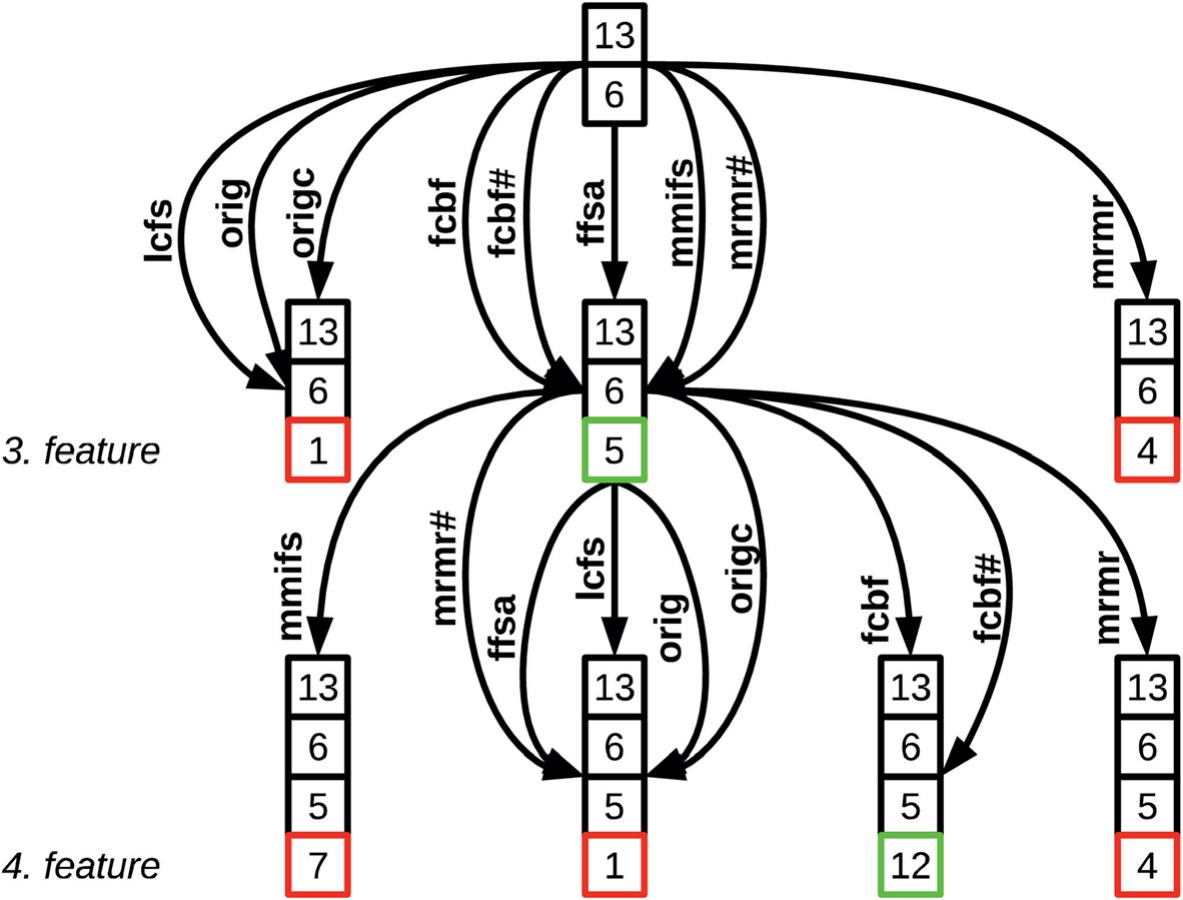
The proposed algorithm is represented by an operation graph, illustrating two sequential steps. It constructs candidate model configurations and selects the feature that results in the smallest estimation error or highest accuracy in the actual extension. The graph nodes are labeled with feature indices represented as numbers and enclosed in frames of different colors. The color variations signify the state of the examined variables. Specifically, features with black frames represent the already selected feature set in the current state, while features with colorful frames (red, green) are the candidate features in the set. The green-framed feature indicates the best feature, which exhibits the smallest estimation error or the highest accuracy compared to other potential variables enclosed in red frames. The directed edges in the graph represent transitions between different states of feature subsets. These subsets correspond to various predetermined feature selection methods employed in this context

Figure 6 illustrates the operation of the proposed algorithm on the Housing dataset [36].

The AHFS algorithm presents a novel approach to feature selection by combining existing supervised techniques and emphasizing adaptivity. Its hybrid nature enables the integration of various feature selection methods and metrics as well. By leveraging the applied learning model and incorporating adaptivity, the AHFS algorithm offers a promising, integrated solution for accurate model building through feature selection. The algorithm demonstrates suitability for real-world applications, including the screening of mental health conditions, due to its robustness and effectiveness in feature selection. Further details can be found in Supplementary 1.4.3

## Results

### Performance of the models

**Fig. 7:**
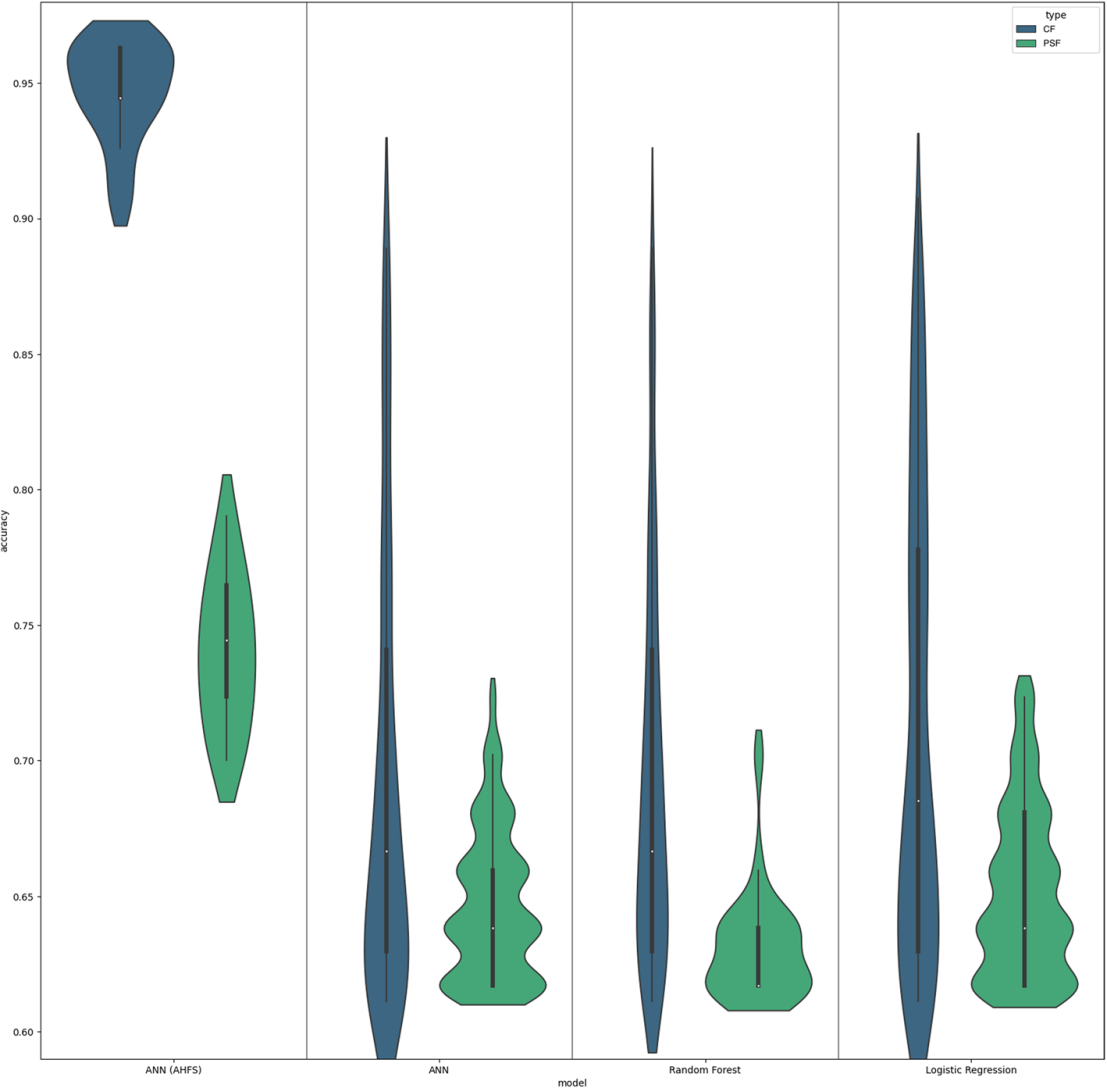
Results of the three algorithms on AHFS and CFSS. Within the Clique Forming Feature Selection (CFFS) framework, models were selected based on achieving at least 60% accuracy. In the USD, Logistic Regression saw 942 models exceed this threshold, while the HUHD had 559. For Random Forest, the numbers were 42 in the USD and 811 in the HUHD. Neural networks had 868 successful models in the USD and 634 in the HUHD. In contrast, the Adaptive Hybrid Feature Selection (AHFS) conducted 20 independent runs, each involving 20 iterative steps, ultimately identifying 400 features. The selection focused on pinpointing the most compact and high-performing feature sets, resulting in 20 optimal models per group

The three models-Logistic Regression, Random Forest, and Neural Networks-demonstrated comparable outcomes, as depicted in Fig. 7. The datasets’ models were trained using their own control groups, due to the differences between the datasets. The two datasets’ characteristics were compared using the aggregated SHAP tables. The Results are further detailed, for example, with exact, best-performing models, with different performance metrics, and a comparison with our previous results [15] in Supplementary 2.

### CFFS findings

The way to interpret a SHAP (Shapley Additive Explanations) plot is that, for example, in the case of the first feature, red dots are located on the right side (with positive Shapley values). This means that the higher the value of that particular feature, the more likely the models were to label the subject with a latency or disorder. On average, the farther away the points are from the y-axis, the greater the impact of that feature, and the features are ranked accordingly. From these extracted pieces of information, we can gain insights into which features are important within the given dataset.

#### Logistic regression

**Fig. 8:**
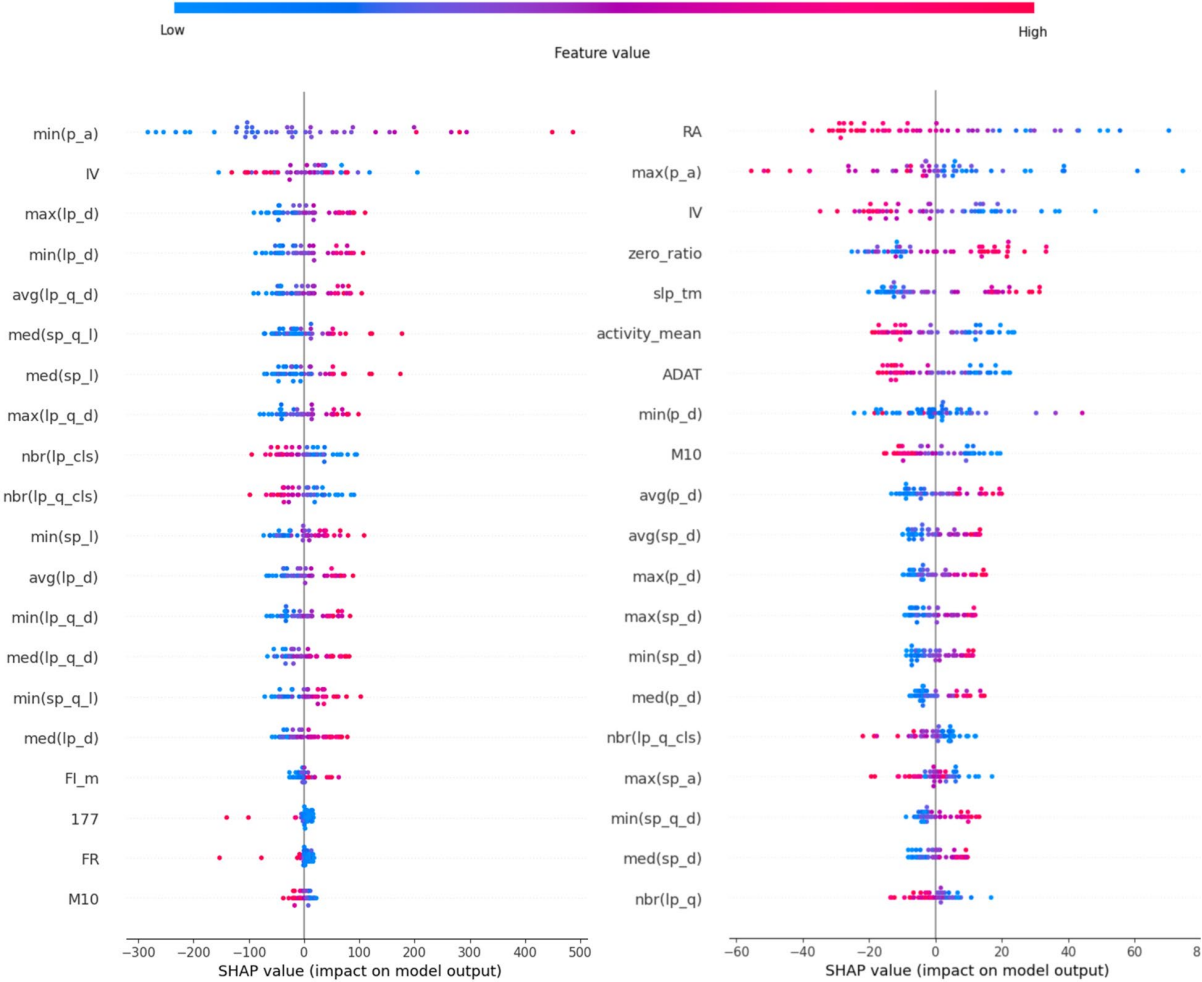
Shapley values of the logistic regression (left: USD, right: HUHD)

As can be seen in Fig. 8, for the USD, sleep-related movements are highly influential, while for the HUHD, in addition to sleep characteristics, RA, IV, ADAT, M10, the mean of activity (all of these metrics’ decrease typical for PSF group), and the ratio of zero values also play a significant role as well. In the USD, features related to the length of small amplitude movements dominated, most specifically the increased values typical for the premorbid latent schizotypy (in the left part of Fig. 8: functions of “sp_l” and “sp_q_l”). Among the large movements, features related to distances increased the most, indicating a decrease in the density of large movements (in the left part of Fig. 8: functions of “lp_d” and “lp_q_d”). In this group, feature IV related to non-sleep activities appeared, but its distribution was not clear, whereas the other features had only a minor contribution.

In the HUHD, the sleep movement-related features are not as relevant as in the other group, but the maximum of the peak amplitudes (decrease of max(p_a)) and the sleep time (increase of slp_tm) played an important role. However, the movement features (in the right part of Fig. 8: functions of “(s)p(_q)_d”) show a decrease in the density (particularly in the small movements).

#### Random forest

**Fig. 9:**
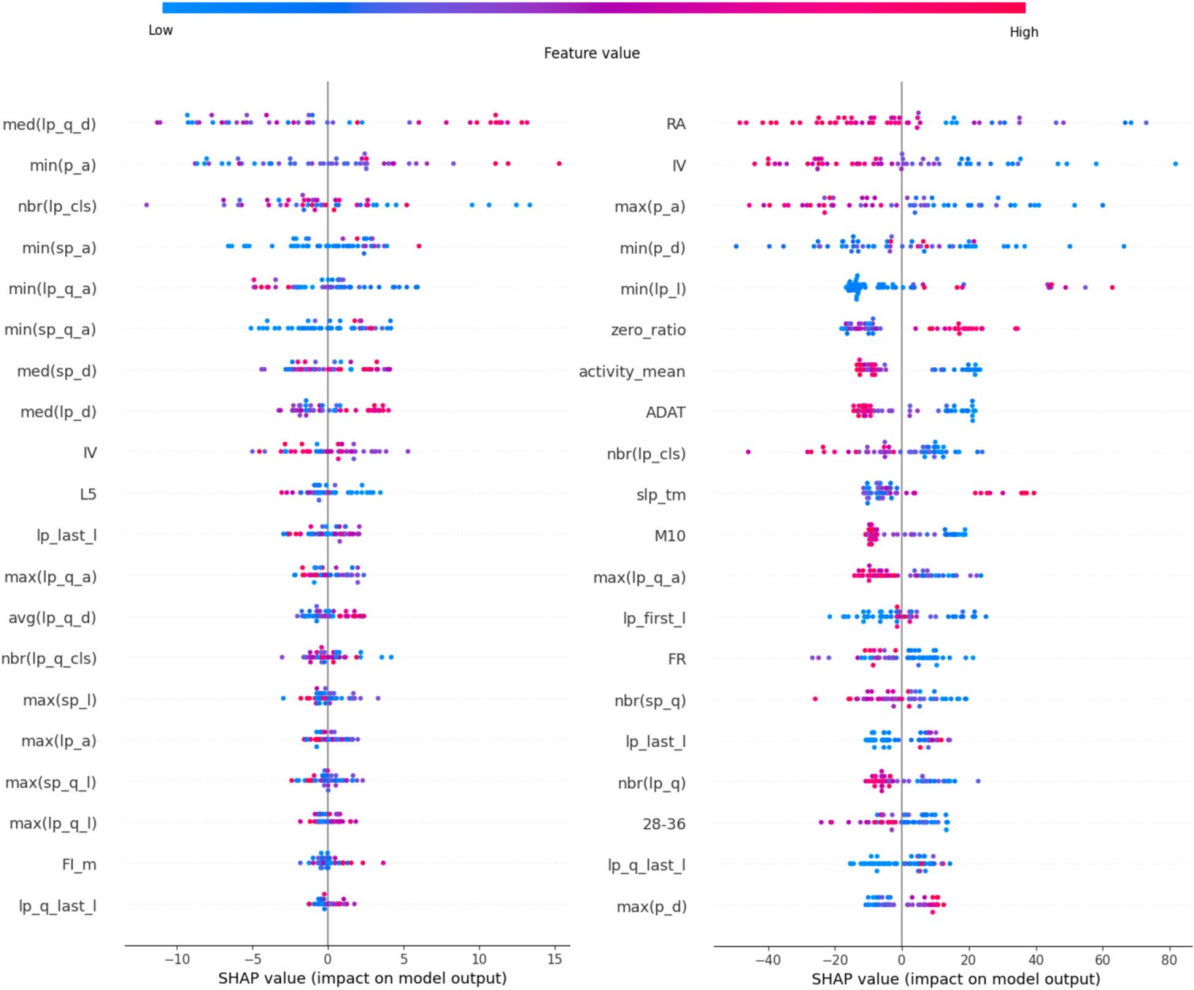
Shapley values of the Random Forest (left: USD, right: HUHD)

Due to the functioning of the Random Forest, it easily captures non-linear relationships, which is evident in Fig. 9 for the PSF group. Even for the most important features, it can be observed that the decisions made cannot be attributed to small or large feature values. The colorings overlap and do not form a continuous transition, so we cannot unambiguously associate a decrease or an increase with these features in relation to the groups. The HUHD shows much smoother transitions along most features, reflecting pronounced symptoms.

#### Artificial Neural Network (ANN)

For the PSF group, sleep movement characteristics dominate, with a focus on both high- and low-amplitude features (“lp_, sp_”), these indicate a decrease in high-amplitude sleep movements, while in the HUHD,the dominance of standard features (IV, RA, M10, ADAT) appear, all of which showed a decrease, the sleep time (“slp_tm”) and the ratio of the zero values to the whole data (“zero_ratio”) showed a significant decrease, and the overall mean of the activity data (“activity mean”) decreased. In the USD, the PSF group, among these features, L5 showed a decrease, while IV was not clear. In the HUHD, several other features were also significant, such as wavelet features indicating reduced regularity, primarily in half-hour resolutions but also for averaged resolutions ranging from 1 to 200 minutes for the CS group compared to its Control Group. Additionally, the fragmentation index calculated from wavelet computations also decreased.

**Fig. 10:**
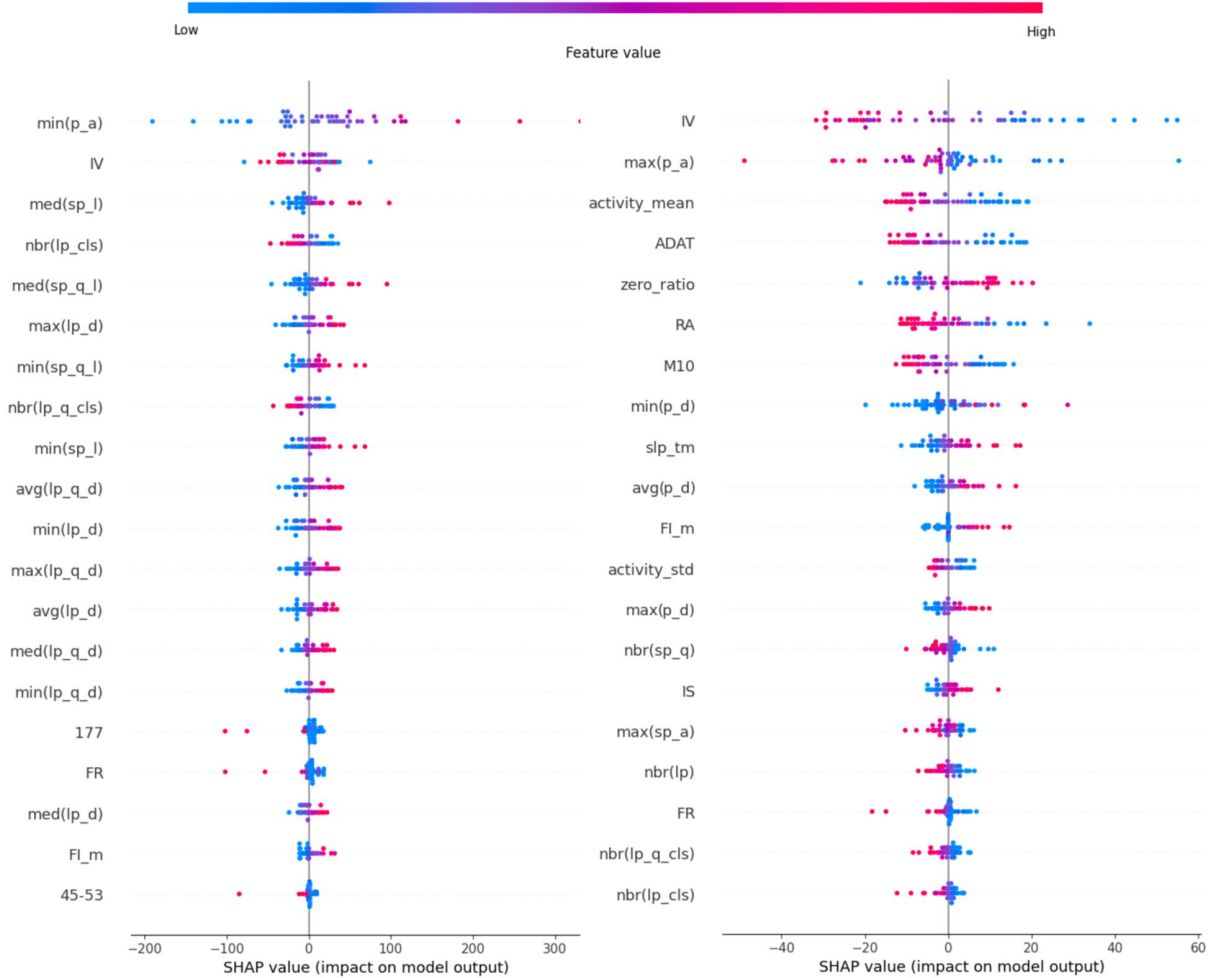
Shapley values of the ANN (left: USD, right: HUHD)

For the PSF group, the first few features are influential, with the amplitude minimum of the sleep movement peaks dominating in the first place. The increased values of this feature were indicative. IV follows, showing a mixed distribution of feature values but playing an important role in the decision-making process. This feature exhibited similar behavior in this group in previous analyses, suggesting that there may be cases where IV, in combination with other features, can indicate the presence of the disorder, regardless of whether its values are low or high. Further research may be warranted on this topic, although we do not delve into it in this study. The remaining features expressed the same patterns as before: an increase in the duration of low-amplitude movements and a decrease in the density of high-amplitude movements, as can be seen in Fig. 10.

In HUHD the CS group, the usual actigraphic features are determinative and typically show decreased values compared to its’ Control Group, besides the increased sleep time and ‘zero_ratio’, the average and variance of overall activity decrease in individuals labeled as patients. The features related to movement during sleep indicate a decrease in their density. Wavelet features indicating structure also show a decrease, suggesting less structured movement during sleep. In the CS group, the density of small and large peaks decreased.

In all three algorithmic analyses (Logistic Regression, Random Forest, and ANN), patterns emerge that highlight different movement and sleep characteristics between the USD and HUHD.

### AHFS findings

**Fig. 11:**
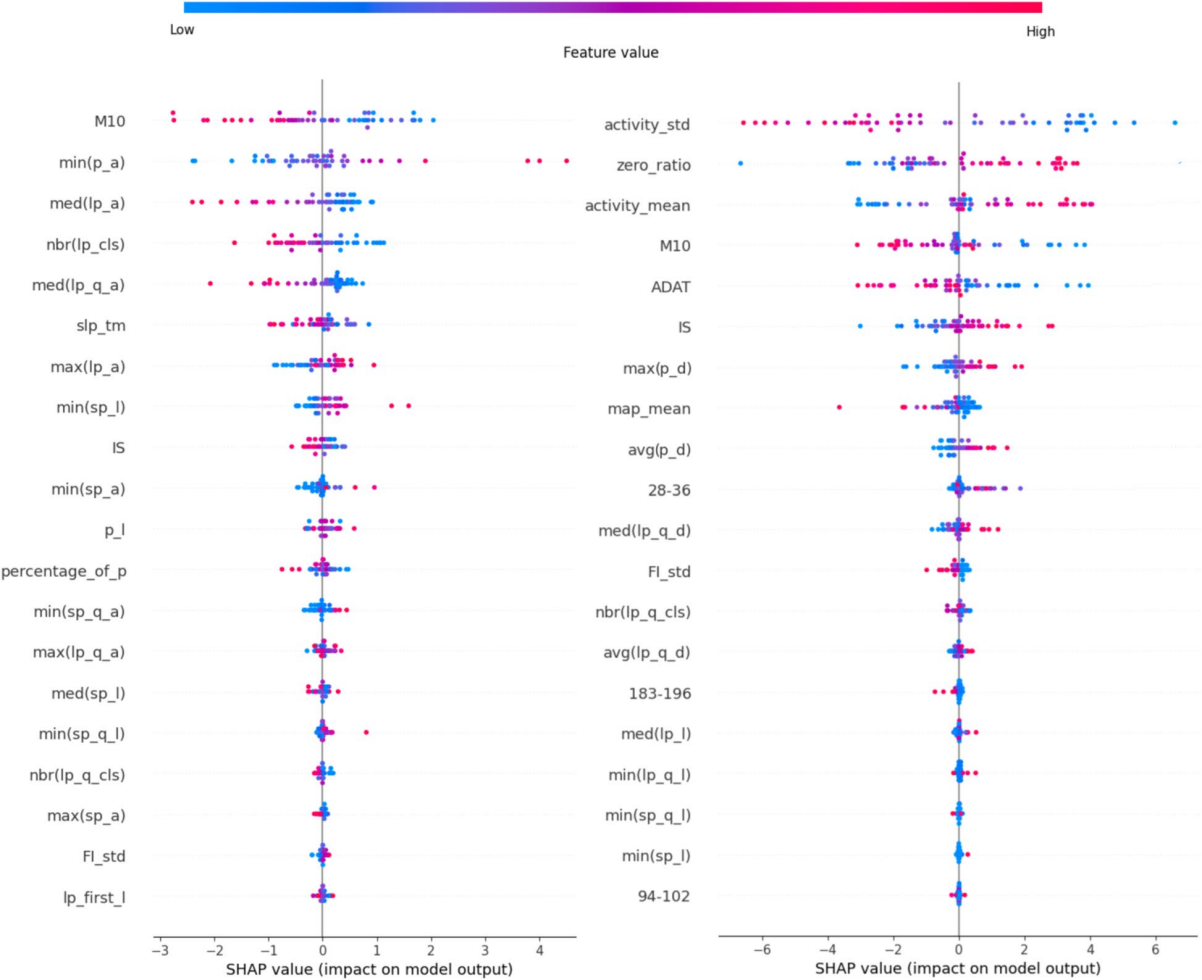
Shapley values of the ANN(AHFS) (left: USD, right: HUHD)

The AHFS employs a variation of the ANN model distinct from the CFFS, necessitated by differences in programming language and environment. An initial candidate feature evaluation model with 8 neurons in its single hidden layer led to overfitting, prompting a reduction to 2 neurons. The algorithm runs 20 iterations to account for ANN’s inherent randomness. Within each iteration, 20 unique models are constructed, each adding one new feature (from a range of 1 to 20). Features are selected based on their performance across various metrics, as described in the AHFS subsection of the Feature Selection chapter. The model with the best accuracy in each iteration is chosen for Shapley value calculations. The Shapley values of these 20 models are then aggregated, similarly to Shapley values from the CFFS, as can be seen in Fig. 11.

The limited number of models and judicious feature selection result in a more tapered “funnel” distribution of Shapley values. In the PSF group, similar to the previous findings, conventional actigraphy metrics are predominant. The standard deviation, mean, and zero ratio of activity data emerge as leaders. Intriguingly, while the raw data and prior analysis indicated an increase in activity mean for schizophrenia patients (CH group), the AHFS suggests a decrease, presenting a contrasting perspective. Among the sleep movement features, only the maximum and the average peak distance are presented, both with increased values, indicating an overall reduction in movement density. Many features align with the findings of the CFFS method, using a similar ANN algorithm. All of the leading conventional actigraphic features, except for the RA, max, and average peak distance (“p_d”), were listed.

In the PSF group, a large dependency on the sleep movement characteristic remains significant, but some standard actigraphic features (M10, IS) and sleep time exhibit decreased values. Many amplitude-related sleep movement features appeared, but they did not clearly indicate an increase or decrease.

## Limitations

Data in the Norwegian database was stored in a poorly documented format, complicating integration and analysis. This heterogeneity may stem from variations in data collection procedures across different healthcare institutions and systems. The Norwegian database contains data from medicated patients, whereas the Hungarian database includes information from medication-free, predisposed individuals. The observed differences between the two groups may, at least in part, reflect lifestyle differences between hospitalized patients and the university-predisposed group. Daytime activities tend to show greater fluctuations, as they are heavily influenced by individual daily routines and external demands, making them more challenging to analyze. In contrast, nocturnal movements are generally more stable, allowing for the identification of subtle differences between groups with greater precision. However, data from the Norwegian database represented the only available patient group for comparison with our results. This study establishes a foundation for future research using actigraphy to track psychiatric disorders. To enhance comparability and generalizability, future studies should focus on forming more homogeneous groups and controlling for medication effects to better isolate the disorder’s impact on motor activity. Additionally, comparing actigraphic patterns in schizophrenia with those in other psychiatric conditions could offer valuable insights, deepening our understanding of these findings.

## Discussion

In the current research study, we have discovered that the three machine learning algorithms (Logistic Regression, Random Forest, and ANN) produced roughly similar results. In the PSF group, sleep-related movements have a substantial impact, while in the CS group, in addition to sleep characteristics, features like RA, IV, ADAT, M10, the mean activity level (all of which decreased), and the ratio of zero values also play a significant role. In the PSF group, features related to the length of small amplitude movements were dominant, particularly the increased values, along with a decrease in the density of large movements. In the CS group, the features related to movement during sleep indicate a decrease in their density. According to previous research studies, schizophrenia patients show a more structured and less complex pattern of motor activity compared to healthy controls and patients with depression [13]. They exhibit lower physical activity levels, as measured by the M10 parameter (the average activity during the 10 most active hours) [37]. In the present study, we found the same when comparing patients (CS groups) to the PSF group. This comparison is a novelty in the literature, as there is a limited amount of research that employs actigraphic features to distinguish between patients and controls, let alone differentiate between PSF and CS patient groups. Berle et al. [13] could successfully identify differences between patients and controls, as well as between patients with schizophrenia and depression, using IS and IV. Schizophrenic patients exhibited a more structured rhythm, suggesting a potentially more organized or monotonous behavioral pattern. This observation is supported by the increased interdaily stability and reduced intradaily variability found in these patients. However, the same pattern was not evident in the depressed patients, despite their similarly reduced activity level. In contrast, patients with bipolar disorder and depression tend to have more disrupted and fragmented sleep-wake patterns, as reflected by higher levels of intra-daily variability (IV) and lower interdaily stability (IS) in their actigraphic recordings [12, 38]. The increased regularity and rigidity of the rest-activity rhythm in schizophrenia, as indicated by higher IS, may be related to premature aging processes and contribute to the severity of negative symptoms [37]. This is in contrast with the more fragmented rhythms seen in other severe mental disorders, like bipolar disorder [12]. In our previous research study [15], we discovered that certain traits can still be discerned even in individuals with a lower susceptibility to schizophrenia or bipolar disorder. Our results showed an overall decrease in daily activity within the two susceptibility groups, which is consistent with existing literature.

In the present study, we found that the altered movement characteristics in the two groups show a specific pattern. Our findings might suggest that during the prodromal phase of schizophrenia, sleep disturbance may play an essential role in the onset of the illness. This has been confirmed by [39], they found that sleep disturbance was one of the most common prodromal symptoms; it was present in 77% of the cases. Our findings indicate that once schizophrenia has developed, daytime activity patterns may also become significant in understanding the symptoms and progression of the illness. Further research is needed to investigate how the pattern changes over time. Sleep and circadian rhythm disruption are prevalent in schizophrenia and have been identified as a potential pathophysiological component of the disease [40–42]. These sleep disturbances include increases in sleep latency, reductions in total sleep time, sleep efficiency, REM sleep latency, and circadian rhythm desynchronization [43]. Importantly, these actigraphic differences appear to be more pronounced in residential schizophrenia patients compared to outpatients, suggesting the treatment setting and illness severity may modulate the sleep-wake and activity disturbances [37]. Although it cannot be excluded that the observed disparity between the two groups in the present study might be attributable to the extrapyramidal (parkinsonian-like) side effects of medication, while the CS group was under medication, the PSF group was not, potentially introducing variability in the observed patterns.

The actigraphy and machine learning combination used in this study represents an innovative methodology that can operate effectively even with small datasets. By utilizing machine learning algorithms, we not only gain a complex overview of the different stages of schizophrenia but also open the door to discovering new relationships within the data. Since each model selects the relevant features independently, rather than relying on the researcher’s preconceptions, the results are not constrained by the researcher’s subjective expectations. This enables the identification of previously unnoticed correlations that might remain hidden when using traditional statistical tests. Machine learning, therefore, enhances the accuracy of defining disease stages and contributes to the understanding of new, yet-to-be-discovered biological and clinical connections related to psychiatric disorders.

In our research, we found that sleep-related actigraphic features may play a role in schizophrenia during its prodromal phase. After the illness develops, daytime activity patterns may also be important for understanding its symptoms and progression. This implies that disruptions in the circadian rhythm could serve as early indicators of the risk of developing schizophrenia. Monitoring sleep disturbances and patterns may be an essential tool in the early detection and prevention of schizophrenia. Also, identifying patterns or changes in daytime activity that correlate with the severity of schizophrenia symptoms is crucial. This may allow for personalized treatment and the timing of interventions. It can be assumed that circadian rhythms and daily activity play complex roles in the development and progression of schizophrenia. These findings could have implications for the diagnosis, treatment, and of the disorder. Further research is necessary to explore the specific effects of actigraphic features in latent, early, prodromal-phase schizophrenia and their potential clinical applications, to gain a more precise understanding of these relationships, and to explore how addressing sleep disturbances and daytime activity management can impact the course of schizophrenia and symptom alleviation.

## Supplementary Information

Supplementary Material 1. The article has several additional parts sourced in the Supplementary Material. There is provided further information about data collection and processing, machine learning algorithms, and other program codes, and more details about the findings

## Data Availability

• Data from the University of Szeged: The previous study provided the used data set [20]. The 42 10-day-long raw triaxial acceleration 615 signals are downloadable from Figshare under a CC-BY 4.0 license through the following DOI: 10.6084/m9.figshare.16437684. • Data from Haukeland University Hospital: The dataset was provided by Jakobsen et al. The dataset available from https://datasets.simula.no/psykose/. The license for this dataset is Creative Commons Attribution- NonCommercial 4.0 International [44].
