# Supplementary material for: The two ends of the spectrum: comparing chronic schizophrenia and premorbid latent schizotypy by actigraphy
Source: BMC Psychiatry. 2025 May 24;25:531. doi: 10.1186/s12888-025-06971-5 (PMC12102934; doi:10.1186/s12888-025-06971-5)
Supplement: Supplementary file 1 — Supplementary Material 1. The article has several additional parts sourced in the Supplementary Material. There is provided further information about data collection and processing, machine learning algorithms, and other program codes, and more details about the findings [file 12888_2025_6971_MOESM1_ESM.zip › Supplementary Materials/Supplementary Materials legends.docx]

The following text furnishes an overview of the supplementary materials.

“Table S1”: This table comprises data pertaining to the sequences that were eliminated from the Norwegian dataset. The timestamps included in the actigraphy data of each participant, which were supplied by Jakobsen et al., indicate which data points our filtering method identified as

damaged. The column labeled "name" contains the original patient ID, where "psykose_patient_" denotes patients with chronic schizophrenia (CS) and "psykose_control_" represents patients under control (C). The beginning and ending dates and times are stored in the "timestamp_beginning" and "timestamp_end" columns, respectively, to identify the damaged sequences.

“Table S2”: This table presents the actigraphy data extracted from the Szeged dataset subsequent to the data cleansing process. The actigraphy data was systematically categorized into distinct days, with each day being numbered within the first twenty-four hours. The usable day numbers are presented in this table, and the participant IDs are listed in the "Index" column (where "Ei_" represents the participants, "_1" signifies the control group, "_3" denotes the PSF schizotypy group, and "_2" signifies the bipolar group, which is not pertinent to this paper).

“Codes” folder: Detailed description in *Supplementary 3*.
