# Supplementary material for: The two ends of the spectrum: comparing chronic schizophrenia and premorbid latent schizotypy by actigraphy
Source: BMC Psychiatry. 2025 May 24;25:531. doi: 10.1186/s12888-025-06971-5 (PMC12102934; doi:10.1186/s12888-025-06971-5)
Supplement: Supplementary file 1 — Supplementary Material 1. The article has several additional parts sourced in the Supplementary Material. There is provided further information about data collection and processing, machine learning algorithms, and other program codes, and more details about the findings [file 12888_2025_6971_MOESM1_ESM.zip › Supplementary Materials/Supplementary.pdf]

Supplementary

1 Methods

1.1 Haukeland University Hospital Dataset

A study was made by Berle et al.[1] among patients with schizophrenia and depression. The study comprised 23 long-term patients with schizophrenia, 23 patients currently experiencing depression, with five of them (28%) being inpatients, and 32 healthy controls. The schizophrenia group contained 3 females and 19 males with an average age of 46.2 ± 10.9 years (range 27– 69 years). Every patient in the schizophrenia group was receiving antipsychotic medication. All subjects were living with a chronic serious illness, in a stable phase, but in long-term institutionalization because they were unable to live independently. The mean age at first time of hospitalization was 24.8 ± 9.3 (range 10–52 years). They were all on antipsychotic maintenance medication. Clinical experts diagnosed the patients using a semi-structured interview based on DSM-IV criteria. The control group consisted of 20 females and 12 males, with a mean age of 38.2 ± 13.0 (range 21– 66 years). Berle et al.[1] collected actigraphic data with a wrist-worn device (Actiwatch, Cambridge Neurotechnology Ltd, England, model AW4, more information in device manual[2]). They used the following variables from actigraphy recordings: total activity, activity night, interdaily stability, intradaily variability, and relative amplitude. They found that motor activity was significantly lower in patients with schizophrenia (153 ± 61, mean ± SD, $p < 0.001$) and depression (187 ± 84, $p < 0.001$) compared to controls (286 ± 80). The actigraph watch records movements with a sampling frequency of 32 Hz and captures movements with 0.05 g resolution. These movements generate a corresponding voltage, which is stored as an activity count in the watch's memory unit. The device utilized a piezoelectric accelerometer to capture the integration of movement intensity, amount, and duration in the x, y, and z axes[3]. The Actiwatch device measures corresponding acceleration on a scale -128 to 128, but preprocessed data were stored. Based on the device manual, the data were filtered in the 3-11 Hz frequency range. After the sleep period identification (details of which can be found in the Feature Engineering subchapter), we assumed that the sleep times longer than 15 hours came from the malfunction of the device and deleted it (deleted periods are provided in the Table S1 supplementary materials). The CS group in the present study is the psychiatric patient population of the \textbf{Haukeland University Hospital Dataset}, sourced from a study by \cite{Berle2010Actigraphic}. Their age ranges from 27 to 69, averaging 46.7 years. Their first hospital treatment occurred, on average, at the age of 24.4, and among them are cases of childhood onset. Thus, on average, they have been living with chronic schizophrenia for 20 years. They all lived in an open but long-term care facility because, according to their caregivers, none of them were suitable for independent living despite their current stable phase of the illness. The overwhelming majority of this population, 87%, is male. In this disease, the course characteristics are generally less favorable among men. Thus, all the disease course data indicate that these patients lived with severe forms of chronic illness and unfavorable disease prospects. They were all under antipsychotic treatment; nearly 40% received clozapine treatment, which is primarily indicated for therapy-resistant cases. Therefore, its use is concentrated among patients with poor prognoses who need institutional placement. Among the patients included in the study, those under clozapine treatment had even more pronounced persistent symptoms in the stable phase, including therapy-resistant cases. The others were either under atypical or conventional antipsychotic treatment. In the original study, the primary actigraphy motion analysis of the patients showed a general reduction in total and nocturnal activity, with an emphasized increase in the regularity of the rest-activity cycle, especially in the group taking clozapine. Despite its advantages, a weakness of the original study is that it did not systematically examine side effects, including those of antipsychotic treatment, particularly pronounced beside conventional drugs but also occurring beside atypical drugs, thus fairly common extrapyramidal side effects. Long-term use of antipsychotics often leads to the development of Parkinsonism, which may be associated with a decrease in the amount of movement, a narrowing of complexity, and stereotyping. This completely corresponds to the actigraphy pattern observed in this patient group. Furthermore, they did not provide the types and doses of the individual drugs used, so this potential and significantly critical correlation cannot be examined from this aspect. They also did not discuss the correlation between primary and negative symptoms and actigraphy indicators. The negative symptom dimension of the basic disease can typically also limit psychomotor behavior along with cognitive dysfunctions (alogia, attention disorder). Spontaneous movements, expressive gestures, and affective reactions may decrease. Physical lethargy and a decline in initiative can be accompanied by social withdrawal and even neglect of personal hygiene. Additionally, it must also be taken into account that those living in the institution do not work and are less active, and their motor behavior is largely determined by the relatively monotonous and stereotypical institutional routine. Based on all this, we can say that the analysis we carried out identified a comprehensive decrease in the average and variability of the amount of movement in chronic patients, which can significantly be related to the patient's long-term and severe basic disease, its symptom pattern, living conditions, and especially the potential side effects of the applied long-term

medication.

1.2 University of Szeged Dataset

The presented information detailed before in the previous study about this dataset[4,5]. The following questionnaires were used in the study for screening: Temperament Evaluation of Memphis, Pisa, Paris, and San Diego Autoquestionnaire (TEMPS-A) is a self-report questionnaire assessing the five affective temperaments: depressed, cyclothymic, irritable, hyperthymic, and anxious ([6]). The questionnaire consists of 110 items for women and 109 for men, with all questions requiring a "yes" or "no" response. The 43-item Hungarian version of the shortened Oxford-Liverpool Inventory of Feelings and Experiences (O-LIFE) was used to assess schizotypal personality traits[7]. The items of the questionnaire can be answered with "yes" or "no". The short version of the questionnaire consists of four scales: Unusual Experiences, Cognitive Disorganization, Introverted Anhedonia, and Impulsive Nonconformity. The Unusual Experiences scale measures the positive trait of schizotypy with items related to perceptual abnormalities, magical thinking, and hallucinations. The Cognitive Disorganization scale includes questions about attention and concentration difficulties, decision-making problems, and social anxiety. The Introverted Anhedonia scale assesses negative schizotypy or schizoid temperament, which can be described as the reduced form of negative symptoms in schizophrenia. The Impulsive Nonconformity scale contains items measuring lack of self-control[7]. The[8] Delusions Inventory (PDI) measures delusions that occur in the normal population. The questionnaire contains 40 questions on different types of delusions (delusions of control, expansive delusions, etc.). Each item of the PDI is rated on a five-point Likert scale under three different aspects: distress (from "not at all distressing" to "very distressing"), preoccupation (from "hardly ever think about it" to "think about it all the time"), and conviction (from "don't believe it's true" to "believe it's absolutely true").

The Clinician Version (SCID-CV) of the Structured Clinical Interview for DSM-5 (SCID-5) was used to diagnose current and lifetime mental disorders ([9]). The SCID-5-CV captures mental disorders such as mood disorders, psychotic disorders, substance use disorders, anxiety disorders, obsessive-compulsive and related disorders, eating disorders, somatic symptom disorders, sleep disorders, externalizing disorders, trauma-related disorders, and stressor-related disorders. The Structured Clinical Interview for DSM-5 Personality Disorders (SCID-5-PD) assesses the personality disorders listed in the DSM-5. Demographic data, family and personal medical history, medications currently being taken, previously diagnosed and treated mental disorders, and family history of symptoms of psychosis or affective spectrum disorder were also recorded. The tools used in the research but not analyzed here include: Agency Attribution Task (developed by our research team); Intentional Binding Task ([10]); Examination of Anomalous Self-Experiences (EASE) ([11]); Discrimination of time intervals (DIS), and production of a single time interval (STT)[12]; the Temperament and Character Inventory-Revised (TCI-R)[13]; the Behavioral Inhibition and Activation System Scales (BIS/BAS Scales)[14]; Morningness–Eveningness Questionnaire[15]; Leuven Affect and Pleasure Scale (LAPS)[16]; the THINC-Integrated Tool (THINC-it) Screening Assessment for Cognitive Dysfunction[17]; Raven's progressive matrices[18]; electroencephalogram (EEG); eye-tracker, assessment of allostatic load (heart rate, blood pressure, cortisol levels, C-reactive protein (CRP), interleukin 6 (IL-6), interleukin 12 (IL-12), total cholesterol; triglyceride levels). Study participants were recruited online via social media platforms and the University of Szeged mailing list. First, participants read general information about the study in a prompt email or online advertisement. They then clicked on a Web link included in the advertisement or email to access the study consent form. After agreeing to participate in the study, they were able to complete a screening package of questionnaires. The subjects were healthy university students, who could not be diagnosed with any current mental disorder, from the first two years of the different faculties of the University of Szeged. In the first step, the intention was to form two homogeneous subgroups in the groups of subjects with low-risk traits for schizophrenic-bipolar spectrum disorders. The test package consisted of four self-completion tests: the PDI-21 to measure delusions, including distress, preoccupation, and conviction[8]; the Hungarian version of the TEMPS-A questionnaire's Cyclothymic Temperament scale[19]; the Hungarian version of the O-LIFE questionnaire measuring schizotypy[7]; and the Hungarian version (unpublished) of the MDQ[20] assessing possible mood disorders. This primary survey was conducted electronically after recruitment through social media channels commonly used by university students.

Inclusion criteria of the Positive Schizotypy Factor group (PSF, presumed to have a latent tendency toward schizotypy): age 18–25 years; written informed consent; abbreviated O-LIFE Unusual Experiences score > median; PDI total score > median. Exclusion criteria: current presence of a primary or secondary mental disorder (SCID-1/2 DSM-5); MDQ scale score of at least 7 for the first question, "yes" for the second question, and "at least a moderate" or "severe problem" for the third question; TEMPS-A Cyclothymia Score > median; current abuse of psychoactive substances; a history of head trauma with permanent loss of consciousness; any physical illness known to affect brain structure; any unstable medical condition that may significantly impair neurocognitive function. Inclusion criteria for Control Group (C): age 18–25 years; written informed consent; abbreviated O-LIFE Unusual Experiences Score < median; PDI total score < median; TEMPS-A Cyclothymia Score < median. Exclusion criteria: current presence of a primary or secondary mental disorder (SCID-1/2 DSM-5); MDQ scale score of at least 7 points for the first question, "yes" for the second question, and "at least a moderate" or "severe problem" for the third question; current abuse of psychoactive substances; a history of head trauma with permanent loss of consciousness; any

physical illness known to affect brain structure; any unstable disease state that may significantly impair neurocognitive function. Based on the online questionnaire, we screened N = 710 students of the University of Szeged. Among the respondents, we selected N = 182 individuals based on the inclusion criteria. Based on the exclusion criteria, N = 57 students were eligible to form the two study groups. However, during the subsequent face-to-face medically structured diagnostic interview, we still had to exclude N = 2 individuals due to acute mental disorder confirmed on the basis of SCID. Based on TEMPS-A, O-LIFE, and PDI values, participants were divided into two groups:

- A schizotypy risk group (hereafter, PSF group, Positive Schizotypy Factor) included those with a PDI-21 total score greater than 10 and an O-LIFE total score greater than 5, but a TEMPS-A Cyclothymia Score less than 12.

- The Control Group included individuals who had relatively low scores on all three questionnaires, with a total score of less than 12 for TEMPS-A Cyclothymia, less than 11 for PDI-21, and less than 6 for O-LIFE.

To ensure comparability, the following groups were included in the study:PSF Group N = 26 students; Group C (control group) N = 29 students. We managed to obtain analyzable actigraphy data from 47 participants (22 male, 25 female): PSF Group (positive schizotypy factor group, presumably showing a latent tendency to schizotypy) NPSF = 22, 11 male and 11 female, mean age MPSF = 26.20, standard deviation of age SDPSF = 2.06.; Group C (control group) NCONTROL = 25, 11 males and 14 females, mean age MCONTROL = 25.42, standard deviation of age SDCONTROL = 1.90. Eighty percent of the subject sample took no medications, 14% took birth control pills, 3% took beta blockers, and 3% took antihistamines. The study was conducted as part of a study entitled “An examination of neurobiological, cognitive and neurophenomenological aspects of the susceptibilities to mood swings or unusual experiences of healthy volunteer students,” and it was approved by the Human Investigation Review Board of the University of Szeged, Albert Szent-Györgyi Clinical Centre, Hungary (No. 267/2018-SZTE) according to its recommendations. All subjects gave written informed consent in accordance with the Declaration of Helsinki, and they were informed of their right to withdraw from the study at any time without providing any explanation. The selected participants received an expense allowance of HUF 15,000 for participation in the entire study, which was obtained through a grant application.

Self-report questionnaires were used for screening participants. The Temperament Evaluation of Memphis, Pisa, Paris, and San Diego Autoquestionnaire (TEMPS-A) is used for measuring depressed, cyclothymic, irritable, hyperthymic, and anxious affective temperament types[6]. The Hungarian version of the shortened Oxford-Liverpool Inventory of Feelings and Experiences (O-LIFE) assesses subclinical schizotypal traits[21]. The Peters et al. Delusions Inventory (PDI) was used for assessing the occurrence and intensity of delusional thinking[8]. The Mood Disorder Questionnaire assesses manic and hypomanic symptoms of bipolar disorder[20]. The Clinician Version (SCID-CV) of the Structured Clinical Interview for DSM-5 (SCID-5) was used for identifying currently present or former psychiatric disorders[9]. Some tools were also applied in the research but were not analyzed in this study (for more details, see[4]).

This earlier study[4] was conducted as part of a larger investigation titled "An examination of neurobiological, cognitive, and neurophenomenological aspects of the susceptibilities to mood swings or unusual experiences of healthy volunteer students" and was approved by the Human Investigation Review Board of the University of Szeged, Albert Szent-Gyorgyi Clinical Centre, Hungary (No. 267/2018-SZTE) in accordance with its recommendations. All participants provided written informed consent in accordance with the Declaration of Helsinki, and they were apprised of their right to disengage from the study at any time and without explanation. The selected participants received a HUF 15,000 expense allowance for the duration of the study, which was attained through a grant application.

In a previous study[5] introduced a compact actigraphy sensor housed in a 3D-printed capsule (41 mm × 16 mm × 11.3 mm, 5.94 g). The sensor included a microcontroller (C8051F410), a 3-axis accelerometer (LIS3DH), a 1 GB flash memory chip, and a quartz clock (accuracy: +/- 20 ppm). We developed this device due to the high cost of commercial sensors and the need for multiple sensors. Our methodology and results align with market-available actigraphs. The customizable sensor offered sampling rates from 1 to 100 samples per second, with the ability to store measurements for weeks. Using this instrument, we conducted 84 measurements over a 4-month period in 2019, to have enough storing space for the at least 2 weeks measure period per person, 10 samples per second rate was chosen. The preprocessing of the triaxial data was provided in our previous study[4]. Many participants’ data were highly damaged due to wrong device usage or device failure. After manual inspection, we deleted the wrong data (exact days per participant shown in Table S2 Supplementaries). The distribution of the counted usable days is shown on Figure **??**.To replicate the other data format as closely as possible, we used a Proportional Integration Method (PIM) in place of the Zero Crossing Method (ZCM). This method is based on the manuscript for the AW4 Actiwatch device. Subsequently, after applying Butterworth filter with cutoff frequencies of 0.25 and 2.5 Hz on the triaxial data and aggregating to an uniaxial magnitude vector, the maximum values per second were extracted, and these maxima were integrated (summed) over a one-minute epoch.

1.3 Feature Engineering

1.3.1 Wavelet

In our analysis, as described in our previous work[4] we employed the Morlet wavelet as the MW, which is a single-frequency sinusoidal wave enveloped by a Gaussian function. The Morlet wavelet on actigraphy data (Figure 1a), also known as the Gabor wavelet, provides a favorable balance between time and frequency resolution[22] and has been utilized effectively in the analysis of physiological signals[23,24]. Continuous wavelet transform was applied to the recorded activity time series $f(t)$, to quantify the multi scale structure of the actograms. The wavelet map was defined by the correlation coefficients of the time series $f(t)$ and the wavelet function $\Psi(t)$, while $*$ stands for complex conjugate:

$$c(u,s) = \int_{-\infty}^{\infty} f(t)\frac{1}{\sqrt{s}}\Psi*\left(\frac{t-u}{s}\right)dt.$$

The calculated $c(u,s)$ is a two-dimensional function of the translation parameter u, scanning along the original time variable t, and s is a scale parameter ("time window"). In our analysis, the Morlet wavelet, with and , was applied:

$$\Psi(t) = \frac{1}{\sqrt{\pi\gamma_b}}e^{-\frac{t^2}{\gamma_b}}e^{-i\omega_c t},$$

where $\frac{\omega_c}{2\pi}$ corresponds to the center frequency, and γ_b to the bandwidth of the transform. Characteristic activity amplitudes corresponding to the actual time window were calculated according to

$$P(s) = \int_u |c(s,u)|^2 du$$

P(s) was determined from concatenated sleep periods of 5 subsequent nights, and the scale variable s spanned from $1s$ to $200s$ in linear scale. In the analysis the $\omega_c = 10$ and $\gamma_b = 2$ were applied.

Continuous wavelet transform (CWT) is an effective and popular tool in the analysis of various types of time series – such as electrophysiological signals (EEG, ECG), gait pace, or even stock market dynamics and landslide events – that contain both stochastic and deterministic components[25–27]. It provides information about the analysed signals both in the frequency and time domain. Morlet wavelet, Gaussian-enveloped sinusoidal, is considered to offer the best tradeoff between time and frequency resolutions, and it is perhaps the most frequently used kernel in wavelet analysis. Here, we applied CWT to identify stochastically occuring motion patterns during sleep periods, in order to provide additional information to sleep fragmentation index, for the characterization of sleep quality of volunteers and patients[4]. By applying CWA with the Morlet wavelet, we aimed to extract crucial information about the dynamic behavior of the biological system and to identify regions of the time series with prominent structural features, as indicated by high amplitude correlation coefficients in the 2D map (as depicted in Figure 1b).

On a linear scale, the sum squared of correlation coefficients derived from the CWA was calculated at various scale factors extending from 1 minute to 200 minutes. These calculations were applied to five consecutive nights of concatenated sleep periods (Figure 1c and Figure 1d). FR (or integrated structure factor) was defined as the integral of the Structure factor function over the time-window range between 20 min and 100 min.

The analysis of the "structure-factor" profiles revealed the presence of prominent characteristics during the few minutes to hours time windows. In our investigation, therefore, the wavelet-based structure parameters were defined as the integral and standard deviation of these curves within specific time intervals. These intervals were 0–15 minutes, 45–53 minutes, and 117 minutes for PSF subjects. In contrast, the corresponding intervals for patients with developed disorders were 28–36 minutes, 94–102 minutes, and 183–196 minutes. The intervals serve as the identifiers of the extracted features, allowing us to readily identify and refer to these particular time intervals in our analysis. The "map_mean" feature was derived for each group by averaging the values of each time interval to provide a representative measure for each group.

1.4 Feature Selection

1.4.1 Shapley Values

We employed two distinct methods for feature selection, both utilizing machine learning algorithms for learning and prediction during the selection process. To compare the outcomes of these methods, we utilized Shapley values, which have been previously used in game theory and provide a convenient way to compare the results.

Originally used as a proven proposition in game theory, Shapley values determine the contribution of each participant to the outcome of a game. In the context of machine learning, Shapley Additive Explanations (SHAP)[28] applies Shapley values to effectively identify the features with the greatest impact on decision-making. The Shapley values of a given machine learning model are theoretically calculated by observing the model's performance on every subset of features. Based on these

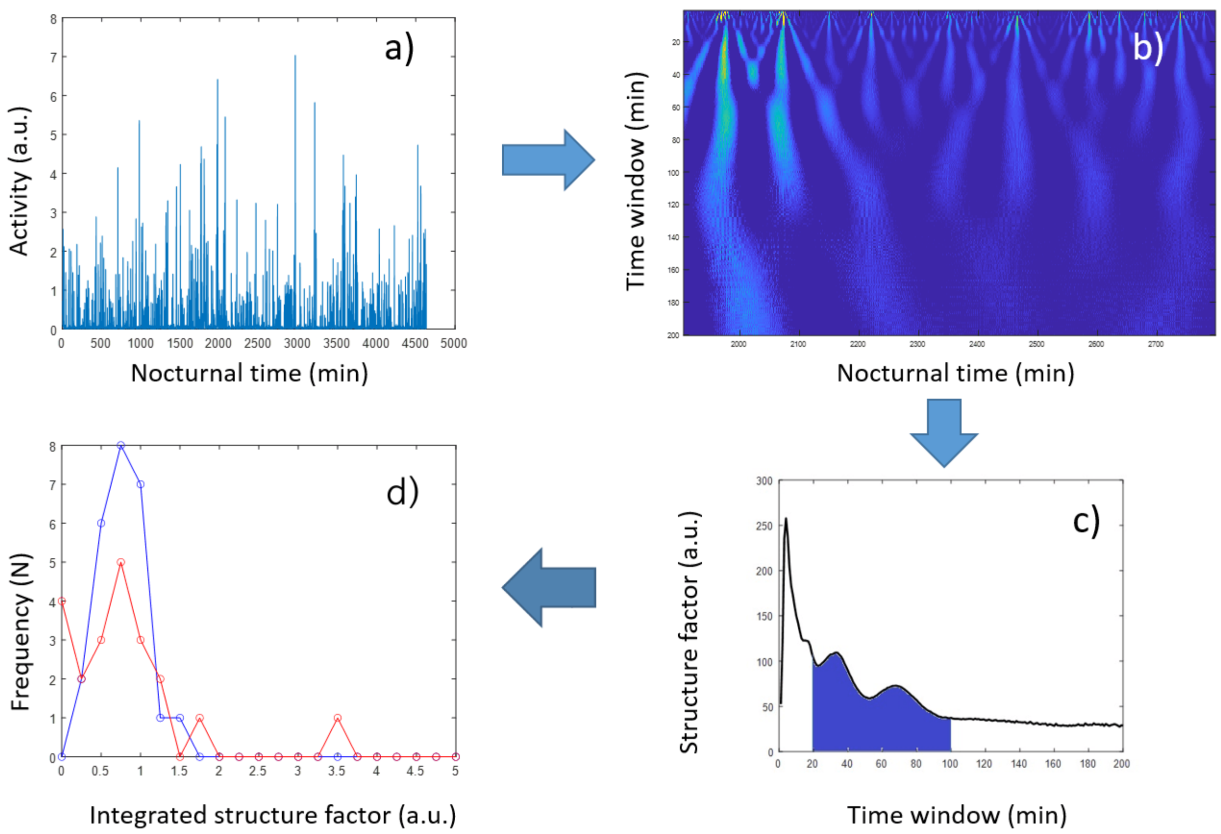

Figure 1. Illustration showing the wavelet-based analysis of nocturnal activity structures. (a) Activities of concatenated sleep periods over the course of five consecutive nights. (b) Correlation-coefficient map of (a)'s time series, as determined by continuous wavelet analysis. (c) Structure parameters derived from the map in (b), as a function of the scale parameter for the 1-200 s time window. (d) Distribution of integrated structure factors (structure_pms) across the two volunteer groups (Control Group (blue) and Positive Schizotypy Factor Group (red)).

observations, SHAP determines the impact of each individual feature on a single prediction. To assess the impact of omitting a particular feature, SHAP replaces its values with random values and makes predictions. The impact is measured by how much the predictions deteriorate without the given feature, for a specific prediction, iteratively over the entire sample. You can interpret it as saying that every prediction is a game where the players are the features and the prediction is the outcome of the game. In practice, the algorithm does not calculate all possible feature combinations but makes assumptions based on a subset of combinations, yielding an approximation of the overall picture.

Each feature has multiple Shapley values, corresponding to the number of decisions made by the model, which in our case aligns with the number of samples in the dataset. In our dataset, each sample corresponds to an individual, providing insights into the effects of features on individual classifications.

Utilizing the SHAP library developed by[28] in Python, we found that the Shapley values of three features evolved as depicted in Figure 2 when using Logistic Regression. In the figure, the Shapley values are plotted on the x-axis. For a given sample, a more positive value indicates a greater contribution of the corresponding feature to being classified as disease-prone by the algorithm. Each point on the plot represents the effect of a single sample on a specific feature, resulting in n points for each feature, where n corresponds to the number of samples (contributors) in this case. The color-coding in the plot indicates the contribution and directionality of particular feature values (small or large values on their respective scales) to the final decision. In this instance, lower values for the mean and standard deviation of daily activity indicate susceptibility to bipolarity. So after the aggregation, a final Shapley plot like Figure 2 robust, and reliable proportional to number of the aggregated models and their performance.

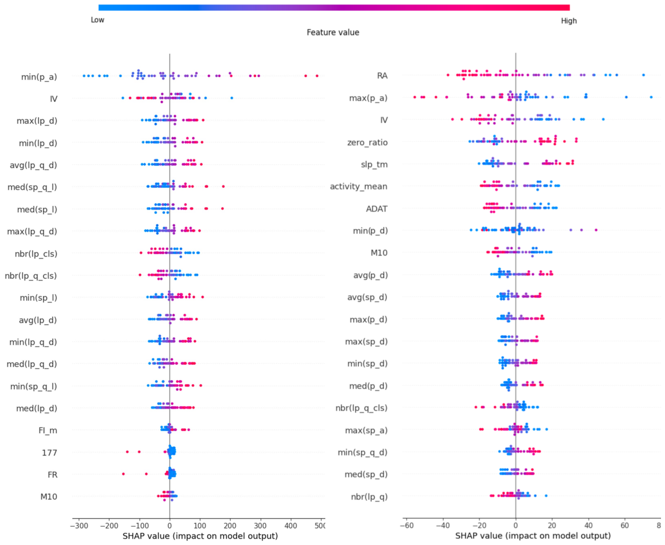

Figure 2

1.4.2 CFFS (Clique Based Feature Selection)

The feature selection procedure, previously presented in[4], was further refined and improved in our study. CFFS uses machine learning algorithms as well to select features. Due to the small datasets and lack of pretrained larger models we are limited to simple machine learning algorithms[29]. In this research, we used the following:

Logistic regression (LR)[30] is a widely used algorithm for binary classification tasks. In our study, we employed LGR for feature selection and classification. The algorithm was initialized with an "l2" penalty metric and the "bilinear" solver. By fitting a linear regression model to the training data and applying a logistic function, LR produces probabilities of instance classification. Logistic regression is a reliable algorithm for interpretable binary classification. With appropriate penalty metrics and solvers, we effectively used LR for feature selection and classification in our study. However, it only works well on a small set of features, not for many features.

Random Forest (RF)[31] is an ensemble learning method where categorization is determined by majority voting based on unpruned classification trees. These trees are grown from random samples of the original data, and at each node, instead of selecting the best split, a random predictor is chosen. Our algorithm utilized 50 trees with a built-in "auto" parameter for limiting the number of features considered at each split. The minimum sample split was set to 2, and there were no restrictions on the maximum depth or number of leaf nodes.

In the following, we want to compare CFFS and AHFS. Since AHFS works in a MATLAB environment and is less malleable, we have tried to integrate the neural network used there into CFFS. However, due to the differences between the two programming languages, this could only be partially done. ANN will serve as a basis for comparing the methods. As the first step in using ANN, we applied a min-max scaler to the data, normalizing it within the range of 0.1 to 0.9. The ANN architecture comprised an input layer, followed by a single hidden layer consisting of 8 neurons that utilized the sigmoid activation function. The output layer consisted of two neurons representing the two classes, employing the softmax activation function. Although the AHFS methodology employed a specific optimizer, we utilized the Adam optimizer in our Python implementation due to the unavailability of the AHFS optimizer in the Python environment.

The CFFS has the following steps. The setps also shown on Figure 3:

- Feature selection based on Welch's test and mutual information metric: Features were examined using Welch's test to assess their statistical significance. Additionally, the mutual information between features was computed. Features were selected if their mutual information was greater than zero and the p-value of the statistical test was less than 0.1, satisfying both conditions simultaneously.

- Pearson correlation computation: From the remaining selected features, pairwise comparisons were made to compute Pearson correlations. This resulted in a complete weighted graph, with the removal of loops and double edges.

- Threshold-based edge deletion: Edges with weights above a certain threshold were deleted from the graph.
- Clique identification: The remaining graph was analyzed to identify cliques, which are complete subgraphs where every node is connected to every other node. A large number of cliques, approximately 10,000 in both cases, were found. From these, a random selection was made, choosing between 3 and 9 cliques of varying sizes. At the PSF group, a 0.2 and the CS group 0.32 correlation threshold was used to extract the 10,000 magnitude number of feature sets, where the random selection was made. The click-forming step must produce that much feature set, because larger sets are not forming with a lowered threshold.

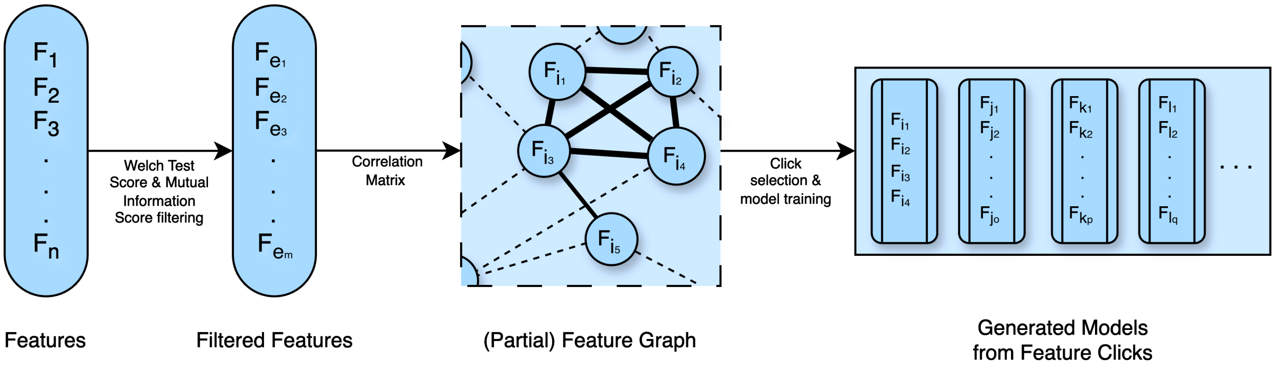

Figure 3. Where F_{1-n} the whole feature set, $F_{e_1-e_m}$ is a feature subset. $F_{i_1-i_4}$ is a 4-element whole subgraph (click) presented in the partial feature graph, giving a visual example of a feature set forming, then models were made with those feature sets.

These cliques, identified through the feature selection process, served as the potential optimal combinations of features for training the three aforementioned learning algorithms. During the learning process, Shapley values were computed, utilizing a 3-fold cross-validation approach. The resulting models were assessed based on their accuracy, with a minimum threshold of 60% set for further analysis.

For each model, a shapely table was generated, consisting of columns representing the features included in the model, rows representing the individual samples, and values corresponding to the Shapley values. By aggregating the corresponding columns from each selected model, a comprehensive understanding of the model's performance within the group was obtained. This aggregation process was conducted for models trained with Logistic Regression, Random Forest, and ANN algorithms, ensuring the elimination of individual outliers and providing a robust depiction of the models' functionality.

By employing this procedure, we were able to select and evaluate models that exhibited satisfactory accuracy levels while leveraging the Shapley values to gain insights into the contributions of different features to the model predictions. This approach enables a more comprehensive analysis of the group and helps establish a reliable framework for further investigations. The utilized machine learning algorithms and all of their parameters and the exact feature selection algorithm are provided in the Supplementary, the codes are in the cffs and machine_learning folders.

1.4.3 AHFS

The Adaptive, Hybrid Feature Selection (AHFS) algorithm[32] introduces a hybrid and adaptive approach to feature selection[33] for machine learning. It combines existing supervised feature selection techniques, each with its own specific evaluation measures, to create a versatile solution. The algorithm incorporates the most frequently used correlation and information-theoretic-based measures, such as MMIFS[34], mRMR[35], LCFS[36], and JMI[37,38], etc., to assess the relationship between features and their information content. This hybridity enables the integration of additional feature selection methods and metrics, making the proposed algorithm adaptable to different scenarios and future scientific results.

The AHFS algorithm employs the widely used Sequential Forward Selection (SFS) technique for feature selection. This method incrementally expands the selected feature set by adding one additional feature at each iteration. Consequently, it is inherent that adaptivity is a key aspect of the AHFS algorithm. It searches not only inside the feature space but at the same time in the space of feature selection techniques and evaluation measures. This adaptivity allows the algorithm to evaluate different feature selection methods at each step, providing a more comprehensive and effective approach.

Figure 4 illustrates the operation of the proposed algorithm on the Housing dataset[39]. The graph nodes are labeled with feature indices represented as numbers and enclosed in frames of different colors. The color variations signify the state of the examined variables. Specifically, features with black frames represent the already selected feature set in the current state, while

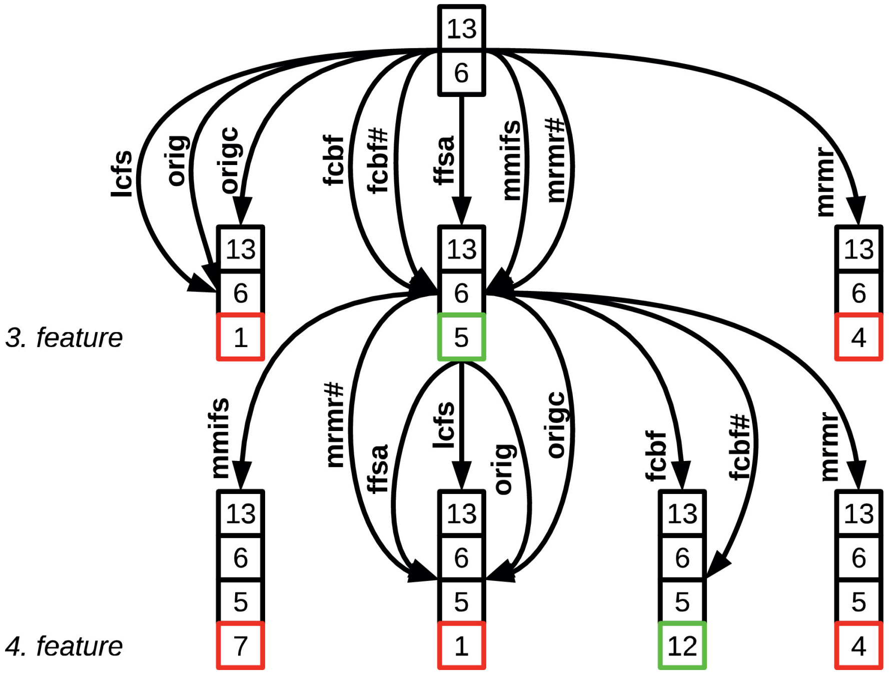

Figure 4. The proposed algorithm is represented by an operation graph, illustrating two sequential steps.

features with colorful frames (red, green) are the candidate features in the set. The green-framed feature indicates the best feature, which exhibits the smallest estimation error or the highest accuracy compared to other potential variables enclosed in red frames. The directed edges in the graph represent transitions between different states of feature subsets. These subsets correspond to various predetermined feature selection methods employed in this context.

In the absence of a universally optimal feature selection algorithm, the AHFS algorithm addresses this limitation by utilizing the applied learning method as an independent evaluation tool. It constructs candidate model configurations and selects the feature that results in the smallest estimation error or highest accuracy in the actual extension. This approach ensures the adaptive selection of features based on the specific learning model used (detailed description of the learning model can be found in the AHFS Findings subsection of the Results section). The algorithm itself yields feature order, accuracy, model error, and concrete models as outcomes, which are essential components of the model's performance evaluation. Consequently, it is inherent that adaptivity is a key aspect of the AHFS algorithm. It searches not only inside the feature space but at the same time in the space of feature selection techniques and evaluation measures. This adaptivity allows the algorithm to evaluate different feature selection methods at each step, providing a more comprehensive and effective approach.

Figure 4 illustrates the operation of the proposed algorithm on the Housing dataset[39]. The graph nodes are labeled with feature indices represented as numbers and enclosed in frames of different colors. The color variations signify the state of the examined variables. Specifically, features with black frames represent the already selected feature set in the current state, while features with colorful frames (red, green) are the candidate features in the set. The green-framed feature indicates the best feature, which exhibits the smallest estimation error or the highest accuracy compared to other potential variables enclosed in red frames. The directed edges in the graph represent transitions between different states of feature subsets. These subsets correspond to various predetermined feature selection methods employed in this context.

In summary, the AHFS algorithm presents a novel approach to feature selection by combining existing supervised techniques and emphasizing adaptivity. Its hybrid nature enables the integration of various feature selection methods and metrics as well. By leveraging the applied learning model and incorporating adaptivity, the AHFS algorithm offers a promising, integrated solution for accurate model building through feature selection. The algorithm demonstrates suitability for real-world applications, including the screening of mental health conditions, due to its robustness and effectiveness in feature selection.

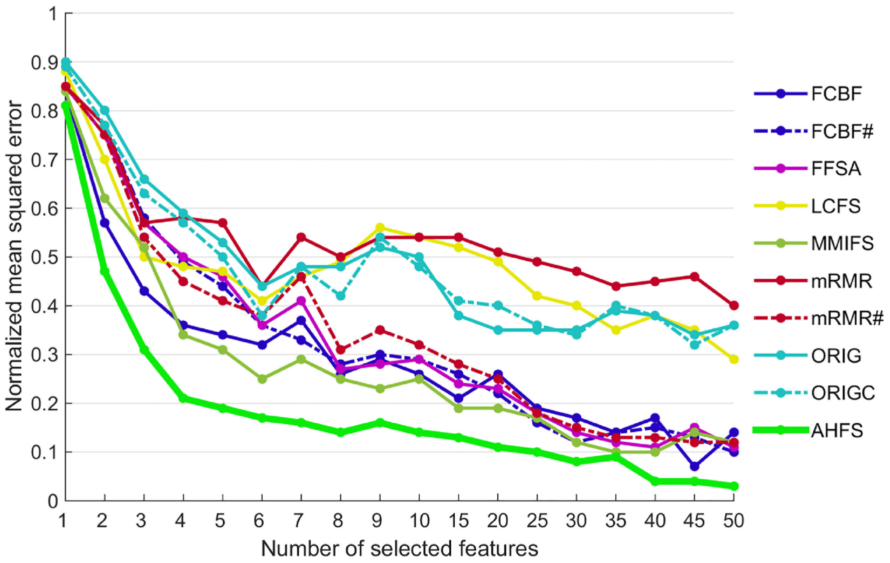

Figure 5. The average performance of both the proposed algorithm and the individual methods.

In Figure 5, the average performance of individual feature selection methods is visualized for each assignment. Each line represents a single feature selection method, with the x-axis indicating the number of features used for model building and the y-axis showing the normalized model error. The overall performance demonstrates that the proposed algorithm generally outperforms the individual methods significantly. Particularly, the most noticeable difference appears in the range of the first 4 to 25 selected features, indicating that the new method identifies the most important features earlier compared to the other methods[32].

2 Results

The models from CFFS were filtered based on performance, with a threshold set at 60% accuracy, resulting in numerical differences. In the PSF group, 942 models performed above the threshold for Logistic Regression, while in the CS group, 559 models met the criteria. For Random Forest, the corresponding numbers were 42 and 811, and for neural networks, they were 868 and 634. On the other hand, within the AHFS framework, 20 independent runs were conducted, each consisting of 20 iterative steps, leading to the discovery of 20x20 features. The selection process involved choosing models with the best performance (the most accurate model) with the optimal number of input features, resulting in a total of 20 models from each group.

As previously mentioned, the two methods, CFFS and AHFS, exhibit notable differences. While CFFS operates on a broader spectrum, encompassing a wide range of models and features to provide a holistic view, AHFS adopts a more targeted approach, aiming to identify the most compact set of top-performing features.

Table 1 displays the best-performing models with their features. To get a comprehensive picture of these models, not just the accuracy but also the precision and recall are presented. These numbers show different metrics of a given model by various relations of the True and False Positive $(TP, FP)$and True and False Negative (TN, FN) predictions. Accuracy (Acc.) is calculated by $\frac{TP+TN}{TP+FN+TN+FN}$, precision (Prec.) is equal to $\frac{TP}{TP+FP}$, and the recall (Rec.) is $\frac{TP}{TP+FN}$. Compared to our previous study on this topic[4] and the same dataset (PSF group), the Mean (M) accuracy and Standard Deviation (SD) have reasonably improved. The Logistic Regression went from (M = 0.6317, SD = 0.044) to (M = 0.6513, SD = 0.0313), and the Random Forest from (M = 0.6103, SD = 0.035) to (M = 0.6309, SD = 0.0197), which is around a 2% increase in accuracy alongside a significant decrease in SD. Regarding maximum accuracy, we did not find better models, presumably due to the stochastic properties of the method (the random selection of the found cliques). The AHFS achieved an impressive accuracy (M = 0.7401, SD = 0.0271) compared to the other algorithms. To get a better, holistic picture, two correlation tables of all the appearing features in the shown on Figure 7 .

CS group					PSF group				
Model	Features	Acc.	Prec.	Rec.	Model	Features	Acc.	Prec.	Rec.

Logistic Regression	ADAT, p_l, nbr(sp), 28-36, min(s_l), IV	0.907	0.87	0.909	Logistic Regression	nbr(lp_cls), 177, max(lp_d), med(sp_l)	0.723	0.714	0.682
	activity_mean, nbr(lp_q_cls)), p_l, min(s_l), min(lp_q_l), IV	0.907	0.87	0.909		max(lp_q_d), min(p_a), nbr(lp_q_cls))	0.723	0.714	0.682
	ADAT, nbr(lp_q_cls)), p_l, min(s_l), min(lp_q_l), IV	0.907	0.87	0.909		med(sp_q_l), min(lp_d), 177, nbr(lp_q_cls))	0.723	0.714	0.682
Random Forest	nbr(lp_q_cls)), p_l, 94-102, M10, min(s_l), IV	0.907	0.947	0.818	Random Forest	min(p_a), max(sp_l), nbr(lp_cls), IV, med(lp_q_d)	0.66	0.625	0.682
	max(p_a), activity_mean, IS, med(sp_l)	0.907	0.947	0.818		min(p_a), nbr(lp_cls), max(lp_q_a)	0.702	0.682	0.682
	max(p_a), activity_mean, nbr(sp), IS	0.907	1	0.773		min(p_a), nbr(lp_cls), max(lp_q_a)	0.702	0.682	0.682
ANN	ADAT, p_l, nbr(sp_q), FR, min(s_l), IV	0.907	0.87	0.909	ANN	med(sp_q_l), min(lp_d), min(p_a),45-53	0.723	0.696	0.727
	ADAT, lp_q_first_l, nbr(sp_q), 28-36, min(s_l), IV	0.907	0.947	0.818		min(p_a), FR, min(lp_q_d), med(sp_l)	0.723	0.696	0.727
	ADAT, nbr(lp_q_cls)), lp_q_first_l, FR, min(s_l), IV	0.907	0.947	0.818		med(sp_q_l), max(lp_q_d), 45-53, nbr(lp_q_cls))	0.723	0.714	0.682

ANN(AHFS)	activity_std,<br>28-36,<br>ADAT,<br>activity_mean,<br>zero_ratio,<br>IS,<br>map_mean,<br>M10,<br>med(lp_q_d)	0.963	0.963	0.952	ANN(AHFS)	min(p_a),<br>med(lp_a),<br>min(sp_a),<br>M10,<br>nbr(lp_cls),<br>med(sp_l),<br>max(lp_a),<br>slp_tm,<br>min(sp_q_a),<br>IS,<br>min(sp_l),<br>p_l,<br>med(lp_q_a),<br>min(sp_q_l),<br>percentage_of_bigger	0.7903	0.749	0.842
	activity_std,<br>activity_mean,<br>28-36,<br>zero_ratio,<br>ADAT,<br>IS,<br>map_mean,<br>M10,<br>med(lp_q_d),<br>183-196	0.963	0.963	0.952		min(p_a),<br>percentage_of_bigger,<br>M10,<br>min(sp_a),<br>med(lp_a),<br>nbr(lp_cls),<br>min(sp_q_a),<br>med(lp_q_a),<br>IS,<br>min(sp_l)	0.7681	0.833	0.501
	activity_std,<br>28-36,<br>ADAT,<br>M10,<br>activity_mean,<br>zero_ratio,<br>IS,<br>map_mean,<br>max(s_l),<br>avg(s_l),<br>min(lp_q_l)	0.963	1	0.911		min(sp_a),<br>med(lp_a),<br>nbr(lp_cls),<br>med(lp_q_a),<br>M10,<br>max(lp_a),<br>max(lp_q_a),<br>med(sp_l),<br>min(sp_q_a),<br>max(sp_a),<br>nbr(lp_q_cls)),<br>med(sp_q_l),<br>IV,<br>IS,<br>min(p_a),<br>slp_tm,<br>min(sp_l),<br>min(sp_q_l),<br>L5	0.7875	0.715	0.822

Table 1. Best-performing models of each algorithm in the two examined groups. Each model represented with their features, and the three performing metrics(Accuracy, Precision, Recall)

	Statistic	Values (University of Szeged Dataset)	Values (Haukeland University Hospital Dataset)
0	Chi-Square Statistic	130.389394	113.135117
1	Chi-Square P-value	0.112906	0.787385
2	Mutual Information Score	3.503430	3.481834

Table 2. Caption

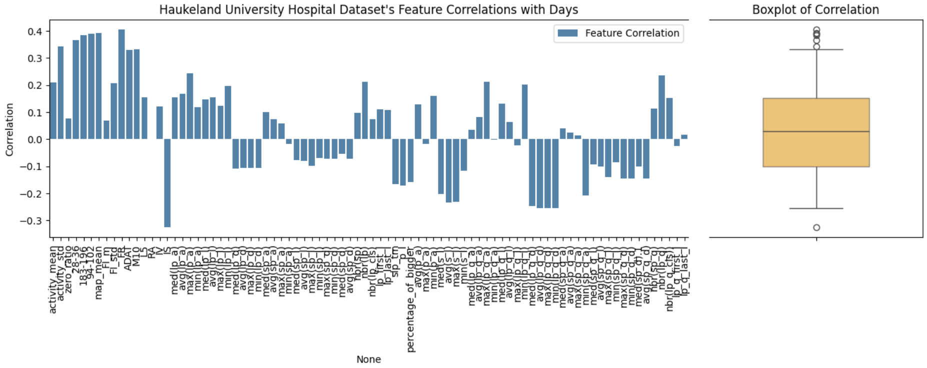

(a) Correlations of the Haukeland University Hospital Dataset features which are appeard at least ones in the final

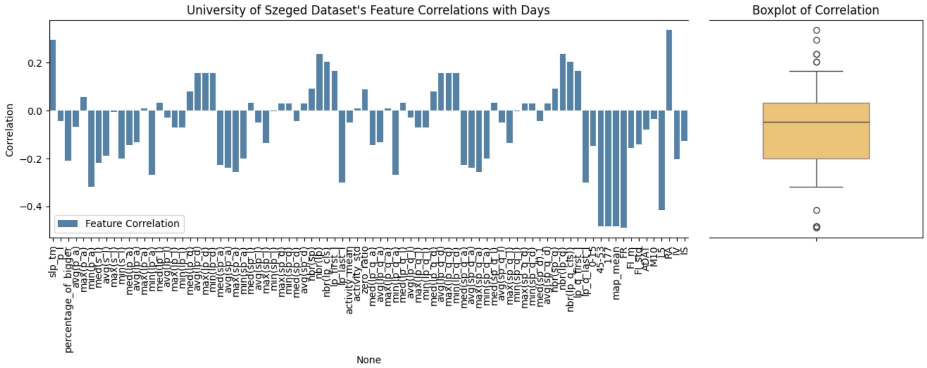

(b) Relative importance scores of the University of Szeged dataset.

Figure 6. Pearson correlations of the feature values and the number of days of raw data each participant has.

In Figure6 can be seen the Pearson correlation of the feaure values and the number of days of raw data had of the participans. Since some cases a mediate correlation of the data length (number of used days) and feautes are presented, we analyzed whether the number of days of data collection had any effect on the predictions made by the machine learning models. The models' predictions, that we used in the analysis are saved, so each sample had several predictions from different models, always in the same order. To summarize these predictions, we took the values corresponding to the same sample across all models, summed them up, and then divided by the number of models. This resulted in an average prediction for each sample, reflecting the overall tendency of the models.

Once we obtained these average predictions, we compared them to the number of days the data was collected. To determine if there was any relationship between them we performed statistical tests for independence. The chi-square test suggested no significant dependence between the two variables, while mutual information analysis showed only a small amount of shared information on both dataset, as it show in Table2.

These results suggest that while some features derived from the raw data might have been influenced by the number of days, this influence did not translate into an effect on the final machine learning predictions. The models appeared to have learned patterns independently of data length, meaning the number of days did not introduce unintended bias into the results.

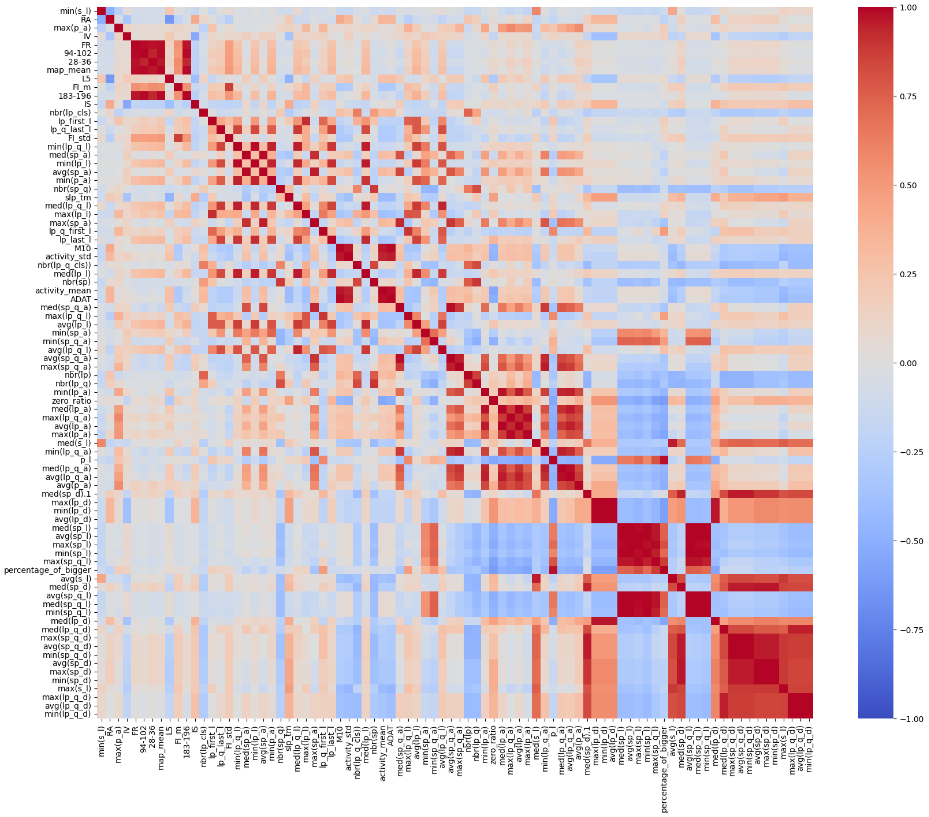

(a) Correlations of the CS group's features which are appeard at least ones in the final

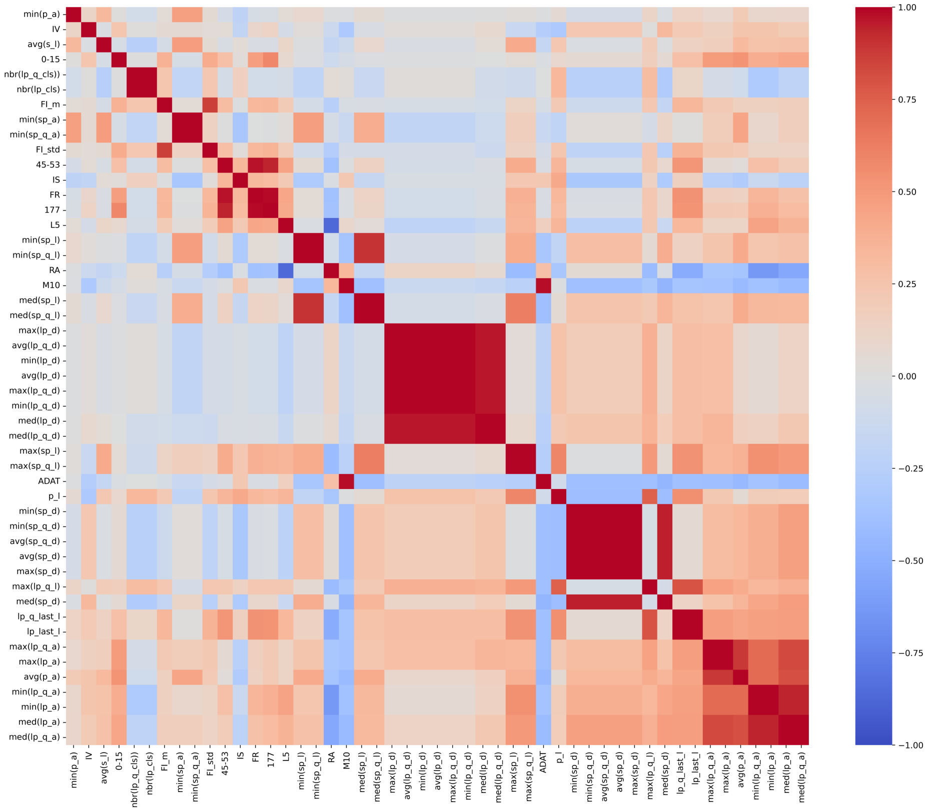

(b) Relative importance scores of the PSF group.

Figure 7. Pearson correlations tables

3 Leave-One-Out training for robustness

For robustness, we tested the performance of each model using Leave-One-Out cross-validation method as well. The metrics were evaluated by storing each prediction until all sample get their value, then the prediction values get compared to the original class labels, resulting in Figure 8, Figure 9 and Figure 10. As the metrics shows, many cases got better performing models, so we can be sure the robustness of the models.

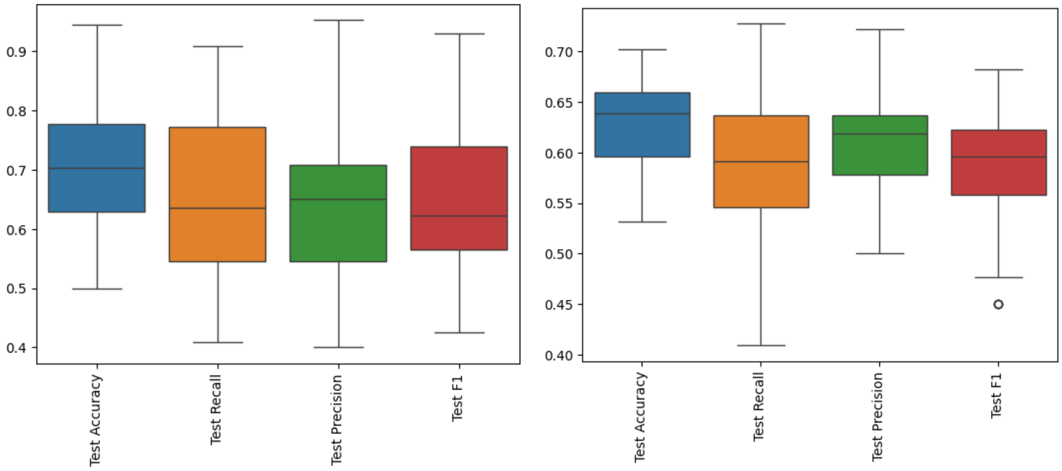

(a) Test result metrics of Leave One Out cross-validation on CS and it's control group

(b) Test result metrics of Leave One Out cross-validation on PSF and it's control group

Figure 8. LLO metrics of Logistic Regression models

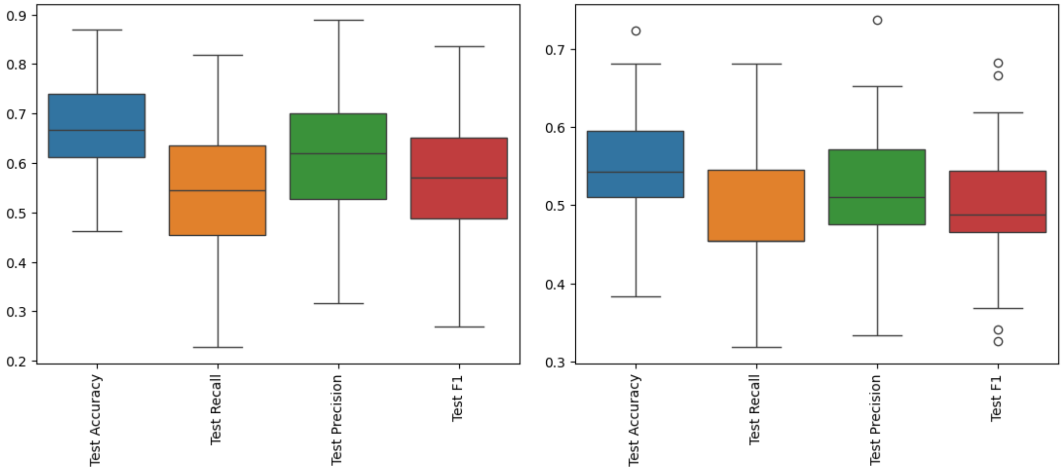

(a) Test result metrics of Leave One Out cross-validation on CS and it's control group

(b) Test result metrics of Leave One Out cross-validation on PSF and it's control group

Figure 9. LLO metrics of Random Forest models

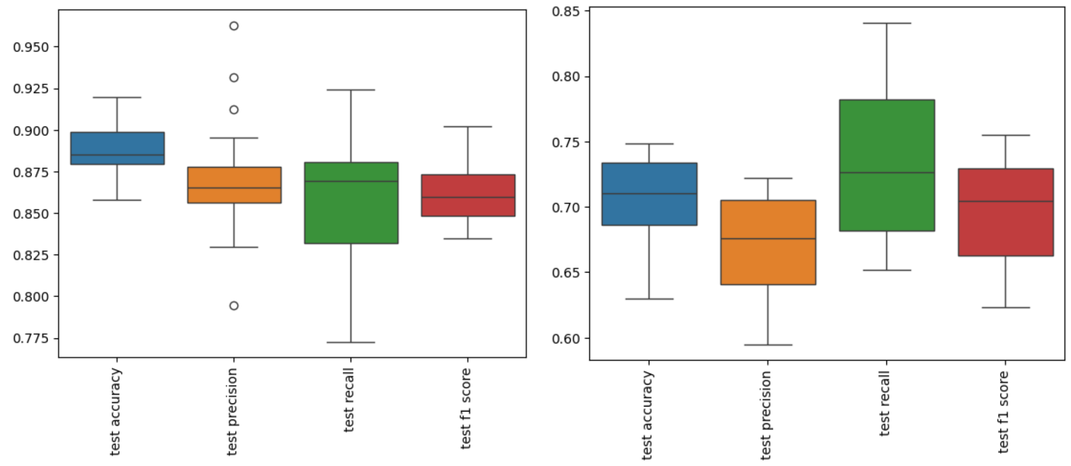

(a) Test result metrics of Leave One Out cross-validation on CS and it's control group

(b) Test result metrics of Leave One Out cross-validation on PSF and it's control group

Figure 10. LLO metrics of ANN models

4 Program Codes

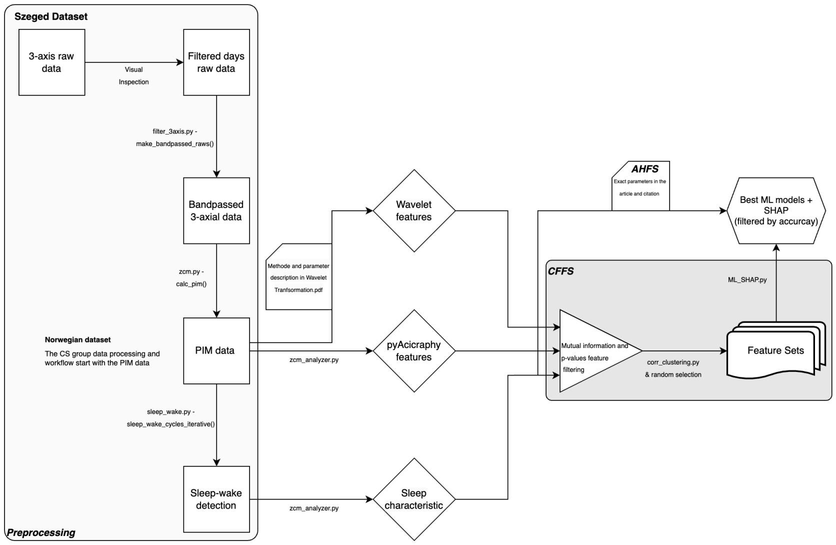

Figure 11. The proposed algorithm is represented by an operation graph, illustrating two sequential steps.

In this section we are provide details about the used program codes and utilizated parameters. The Figure 11 is a flowchart about the exact useage of the provided codes. Blew there is a short description about each script:

- `corr_clustering.py`: This script is designed for clustering correlated data. It appears to use statistical methods to identify and group highly correlated features, enabling efficient analysis of the dataset. It is used in the **CFFS (Correlation and Feature Filtering Strategy)** step to reduce redundancy by clustering highly correlated features together, thus ensuring the selected feature set is more informative. The script uses correlation metrics to calculate relationships between variables and employs a clustering algorithm to group similar features. To use this script, provide a CSV file with data to be analyzed (where columns are features to be clustered) as input. The output is a set of clustered groups of features, which can help in reducing dimensionality or identifying similar behaviors. Clustering parameters, such as the correlation threshold, can be customized to control the grouping.

- `networkx_graph_drawer.py`: This script provides a utility to visualize graphs using NetworkX. It is particularly useful for visualizing relationships and clusters identified in correlation or other data structures. The script uses NetworkX to create and draw graphs and supports customization of node and edge properties. Input to the script is a graph structure, typically provided in a format compatible with NetworkX (e.g., adjacency list or edge list). The output is a visual representation of the graph, either displayed in a window or saved as an image file. Graph properties like node size, color, edge weight, and layout can be customized.

- `ML_SHAP.py`: This script is focused on using SHAP (SHapley Additive exPlanations) values for model interpretability. It allows users to understand the influence of each feature on the model's predictions. It is used during the **Machine Learning Model Training and Evaluation** step, where it helps to train models on the selected feature sets and interpret the results. The script trains a machine learning model on the provided dataset and computes SHAP values to interpret the model's decision-making process. Input to the script is a dataset in CSV format for training the model, and the output includes SHAP values for each feature along with visualizations of their impact. Model parameters and SHAP visualization settings can be adjusted as needed.

- `calculate_pyacti_values.py`: This script is used to calculate activity values, likely for actigraphy data, to assess physical activity levels over time. It reads actigraphy data and calculates summary statistics or activity metrics. The input is a CSV file containing time-series actigraphy data, and the output includes summary values such as activity counts, averages, or sleep/wake estimations. Parameters such as activity threshold values and time window sizes can be set.

- `filter_3axis.py`: This script filters 3-axis accelerometer data, commonly used in actigraphy or motion sensing. It is used during the **Preprocessing** step to apply bandpass filtering to the raw 3-axial data, removing noise and extracting relevant frequency ranges. The script applies filters to smooth accelerometer data, removing noise and highlighting patterns. Input to the script is a CSV file with 3-axis accelerometer data (X, Y, Z), and the output is filtered accelerometer data that is useful for further analysis. Filter type (e.g., low-pass, high-pass) and cutoff frequency can be specified.

- `zcm_analyzer.py`: This script analyzes time-series data, possibly for identifying patterns like sleep/wake cycles. It is used to extract features such as **pyActigraphy features** and **sleep characteristics** from actigraphy and sleep-wake detection data. The script calculates ZCM or other actigraphy metrics to determine variability in the signal and aggregates ZCM values over specified time intervals. Input to the script is time-series data (e.g., CSV file with raw signals), and the output includes ZCM analysis results, including aggregated values. Parameters such as time window for aggregation and ZCM threshold can be set.

- `zcm_helper.py`: This is a helper module that provides utility functions for data analysis. It contains functions for filtering activity values, detecting events, and aggregating data. This script is used as an import by other scripts such as `zcm_analyzer.py`. It is not intended for direct execution, but its functions include adjustable thresholds and filtering options.

- `zcm.py`: This script serves as the main script to coordinate zero-crossing analysis, possibly integrating functions from `zcm_helper.py` and `zcm_analyzer.py`. It is used to calculate PIM data from the bandpassed 3-axial data in the preprocessing phase. The script provides an interface for full data analysis on given datasets and handles preprocessing, calculation, and visualization steps. Input to the script is time-series datasets to be analyzed, and the output includes results of the actigraphy metric analysis, visualized and/or saved to file.

- `sleep_wake.py`: This script analyzes sleep-wake patterns based on activity data, identifying periods of sleep and wakefulness. It is used in the **Sleep-Wake Detection** step to classify sleep and wake periods from the activity data. Hierarhically . The script uses activity thresholds to classify sleep and wake periods and outputs detailed reports on sleep-wake cycles. Input to the script is CSV files with activity data, typically from actigraphy devices, and the output includes sleep-wake cycle estimations and related statistics. Parameters such as activity thresholds for sleep classification, left/right window sizes for smoothing, and iteration options can be specified.

For more detail, exact parameters and the commented codes see the "*codes*" folder.

References

1. Berle, J. O., Hauge, E. R., Oedegaard, K. J., Holsten, F. & Fasmer, O. B. Actigraphic registration of motor activity reveals a more structured behavioural pattern in schizophrenia than in major depression. *BMC Res. Notes* **3** (2010).

2. The Actiwatch User Manual (2008). Last accessed on 23/01/2024., https://www.salusa.se/Filer/Produktinfo/Aktivitet/TheActiwatchUserManualV7.2.pdf.

3. Jakobsen, P. *et al.* Psykose: A Motor Activity Database of Patients with Schizophrenia. *2020 IEEE 33rd Int. Symp. on Comput. Med. Syst. (CBMS)* (2020).

4. Nagy, Á. *et al.* The Actigraphy-Based Identification of Premorbid Latent Liability of Schizophrenia and Bipolar Disorder. *Sensors* **23**, 958 (2023).

5. Maczák, B., Vadai, G., Dér, A., Szendi, I. & Gingl, Z. Detailed analysis and comparison of different activity metrics. *PLOS ONE* **16**, e0261718 (2021).

6. Akiskal, H. S., Akiskal, K. K., Haykal, R. F., Manning, J. S. & Connor, P. D. Temps-A: progress towards validation of a self-rated clinical version of the Temperament Evaluation of the Memphis, Pisa, Paris, and San Diego Autoquestionnaire. *J. Affect. Disord.* **85**, 3–16 (2005).

7. Mason, O., Linney, Y. & Claridge, G. Short scales for measuring schizotypy. *Schizophr. Res.* (2005).

8. Peters, E. R., Joseph, S. A. & Garety, P. A. Measurement of Delusional Ideation in the Normal Population: Introducing the PDI (Peters et al. Delusions Inventory). *Schizophr. Bull.* **25**, 553–576 (1999).

9. First, M. B. Structured Clinical Interview for the DSM (SCID). *The Encycl. Clin. Psychol.* 1–6 (2015).

10. Haggard, P., Clark, S. & Kalogeras, J. Voluntary action and conscious awareness. *Nat. neuroscience* **5**, 382–385 (2002).

11. Parnas, J. *et al.* Ease: examination of anomalous self-experience. *Psychopathology* **38**, 236–258 (2005).

12. Merchant, H., Zarco, W. & Prado, L. Do we have a common mechanism for measuring time in the hundreds of millisecond range? evidence from multiple-interval timing tasks. *J. neurophysiology* **99**, 939–949 (2008).

13. Cloninger, R. The temperament and character inventory (tci): A guide to its development and use. st. *(No Title)* (1994).

14. Carver, C. S. & White, T. L. Behavioral inhibition, behavioral activation, and affective responses to impending reward and punishment: the bis/bas scales. *J. personality social psychology* **67**, 319 (1994).

15. Horne, J. A. & Ostberg, O. A self-assessment questionnaire to determine morningness-eveningness in human circadian rhythms. *Int. journal chronobiology* **4**, 97–110 (1976).

16. Demyttenaere, K., Mortier, P., Kiekens, G. & Bruffaerts, R. Is there enough “interest in and pleasure in” the concept of depression? the development of the leuven affect and pleasure scale (laps). *CNS spectrums* **24**, 265–274 (2019).

17. McIntyre, R. S. *et al.* The thinc-integrated tool (thinc-it) screening assessment for cognitive dysfunction: validation in patients with major depressive disorder. *The J. clinical psychiatry* **78**, 20938 (2017).

18. Raven, J. C. & Court, J. *Raven’s progressive matrices* (Western Psychological Services Los Angeles, CA, 1938).

19. Rozsa, S. *et al.* Az affektív temperamentum: a temps-a kérdõívvel serzett hazai tapasztalatok. *Psychiatr. Hungarica* **21**, 147–160 (2006).

20. Hirschfeld, R. M. *et al.* Development and Validation of a Screening Instrument for Bipolar Spectrum Disorder: The Mood Disorder Questionnaire. *Am. J. Psychiatry* **157**, 1873–1875 (2000).

21. Kocsis-Bogár, K., Nemes, Z. & Perczel-Forintos, D. Factorial structure of the Hungarian version of Oxford-Liverpool Inventory of Feelings and Experiences and its applicability on the schizophrenia-schizotypy continuum. *Pers. Individ. Differ.* **90**, 130–136 (2016).

22. Gabor, D. Theory of communication. Part 1: The analysis of information. *J. Inst. Electr. Eng. - Part III: Radio Commun. Eng.* **93**, 429–441 (1946).

23. Mallat, S. *A Wavelet Tour of Signal Processing* (Elsevier, 1999).

24. Daugman, J. G. Two-dimensional spectral analysis of cortical receptive field profiles. *Vis. Res.* **20**, 847–856 (1980).

25. Sethi, D., Bharti, S. & Prakash, C. A comprehensive survey on gait analysis: History, parameters, approaches, pose estimation, and future work. *Artif. Intell. Medicine* **129**, 102314, DOI: https://doi.org/10.1016/j.artmed.2022.102314 (2022).

26. Tomás, R., Li, Z., Lopez-Sanchez, J. M., Liu, P. & Singleton, A. Using wavelet tools to analyse seasonal variations from insar time-series data: a case study of the huangtupo landslide. *Landslides* **13**, 437–450, DOI: https://doi.org/10.1007/s10346-015-0589-y (2016).

27. Iranmanesh, S. & Rodriguez-Villegas, E. A 950 nw analog-based data reduction chip for wearable eeg systems in epilepsy. *IEEE J. Solid-State Circuits* **52**, 2362–2373, DOI: 10.1109/JSSC.2017.2720636 (2017).

28. Lundberg, S. M. & Su-In, L. *A Unified Approach to Interpreting Model Predictions*, vol. 30, 4765–4774 (In Proceedings of the Advances in Neural Information Processing Systems, Long Beach, CA, USA, 2017).

29. Raudys, S. & Jain, A. Small sample size effects in statistical pattern recognition: recommendations for practitioners and open problems. In *[1990] Proceedings. 10th International Conference on Pattern Recognition*, vol. i, 417–423 vol.1, DOI: 10.1109/ICPR.1990.118138 (1990).

30. Gasso, G. Logistic regression (2021).

31. Liaw, A. & Wiener, M. Classification and regression by randomforest. *R News* **2**, 18–22 (2022).

32. Viharos, Z. J., Kis, K. B., Fodor, Á. & Büki, M. I. Adaptive, Hybrid Feature Selection (AHFS). *Pattern Recognit.* **116**, 107932 (2021).

33. Chandrashekar, G. & Sahin, F. A survey on feature selection methods. *Comput. Electr. Eng.* **40**, 16–28 (2014).

34. Song, J., Zhu, Z., Scully, P. & Price, C. Modified Mutual Information-based Feature Selection for Intrusion Detection Systems in Decision Tree Learning. *J. Comput.* **9** (2014).

35. Li, Y., Yang, Y., Li, G., Xu, M. & Huang, W. A fault diagnosis scheme for planetary gearboxes using modified multi-scale symbolic dynamic entropy and mRMR feature selection. *Mech. Syst. Signal Process.* **91**, 295–312 (2017).

36. Jiang, S.-y. & Wang, L.-x. Efficient feature selection based on correlation measure between continuous and discrete features. *Inf. Process. Lett.* **116**, 203–215 (2016).

37. Hua Yang, H. & E. Moody, J. Feature Selection Based on Joint Mutual Information. *Proc. international ICSC symposium on advances intelligent data analysis* **23** (1999).

38. Hua Yang, H. & E. Moody, J. Data visualization and feature selection: New algorithms for nongaussian data. *Adv. neural information processing systems* **12** (1999).

39. Bache, K. & Lichman, M. UCI machine learning repository (2013).
